# Surface Properties of Colloidal Quantum-Confined One-Dimensional Lepidocrocite Titanates: Insights into their Ion-Induced Gelation

**DOI:** 10.1021/acs.langmuir.5c02076

**Published:** 2025-09-10

**Authors:** Adam D. Walter, Vanessa R. Morris, Jacob M. Nantz, Timothy F. Niper, Laura Galeano Tirado, Mary Qin Hassig, Abijah Gordon, Tongjie Zhang, Ahmed M. H. Ibrahim, Gregory R. Schwenk, Jairo A. Díaz A., Andrew J. D. Magenau, Christopher Y. Li, Michel W. Barsoum

**Affiliations:** † Department of Materials Science and Engineering, 6527 Drexel University, Philadelphia, Pennsylvania 19104, United States; ‡ Department of Chemical Engineering, 6925 Rochester Institute of Technology, Rochester, New York 14623, United States

## Abstract

The surfaces of 1D layered lepidocrocite-structured titanates (1DLs) are negatively charged due to an oxygen-to-titanium atomic ratio >2. This, and their layered structure, allow for facile ion exchange and high colloidal stability, demonstrated by ζ-potentials of ≈ −85 mV at their unadjusted pH of ≈10.4. This is nearly maintained across a 20 to 70 °C temperature range, with only a slight decrease in stability. The acid resistance of 1DL solids (little dissolution until pH 1) is demonstrated through inductively coupled plasma mass spectrometry. The Fourier transform infrared spectra of the dried 1DLs are also discussed. From a fundamental charge perspective, these materials offer an ion exchange capacity of ≈1.8 mmol/g, nearly *twice* that of highly charged clays or Nafion. As a Brønsted–Lowry base, they readily adsorb protons onto their heterogeneous surfaces, as illustrated by an isothermal adherence to the Freundlich model. 1DLs have two p*K*_a_ values, one at pH ≈10.9 and the other at ≈3.2, and can be protonated to their point of zero charge (≈pH 6.8) before they destabilize. With the understanding of the acid/base properties of 1DLs, cation-stabilized hydrogel-like solids were formed using H^+^, Li^+^, Na^+,^ K^+^, Mg^2+,^ Ca^2+^, Ba^2+^, and Fe^3+^. A gelation mechanism is proposed that relies on cation exchange being the driving force for water removal from between adjacent 1DLs. The rheological properties of the soft H_3_O^+^-cross-linked gel-like solids show a more than 1000-fold increase in the viscosity of the 1DL colloidal suspensions compared to before gelation.

## Graphical abstract

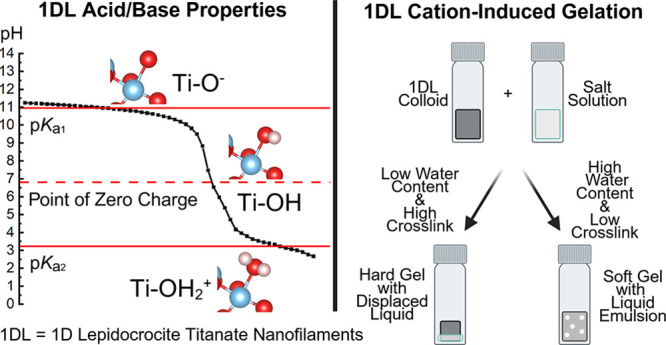

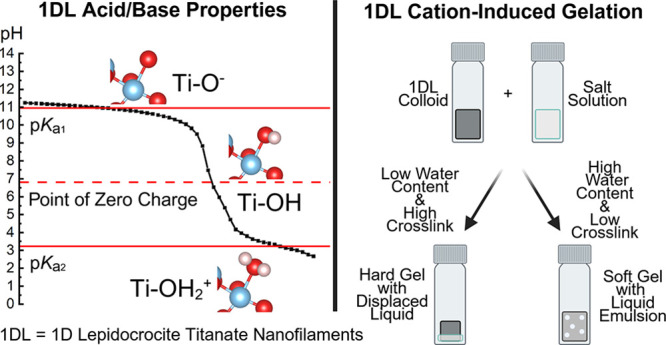

## Introduction

Titania, or TiO_2_, is a ubiquitous commodity oxide used in a wide variety of applications ranging from paint, biomedicine, and catalysis.^1,2^ When synthesized under highly basic or oxygenated environments, layered lepidocrocite titanates (LTs) are formed.^3–5^ These layered LTs are comprised of edge-sharing TiO_6_ octahedra that require cations in the interlayer space to neutralize the negative charge of the oxygen-rich backbone between the layers for charge balance.^3,4,6^ Their cations vary, but are usually comprised of the alkali cations: sodium, Na^+^, potassium, K^+^, for nano-LTs^3,4^ or cesium, Cs^+^, for bulk-LTs.^5^ Sasaki and co-workers pioneered the acid etch method for delaminating bulk Cs-LTs into layered protonic LT sheets,^7^ which are akin to the materials discussed in this work. LTs can be used for a variety of applications, such as dye sensitized solar cells,^8^ batteries,^9^ catalyst supports,^10^ or applied directly as photocatalysts.^11^

The surface chemistry of both TiO_2_ and LTs has been studied widely throughout the literature, both for fundamental and application-driven work. Single-crystal TiO_2_ surfaces are popular for surface modification studies due to the relative simplicity of interpreting the results and the commercial availability of high-quality thin films.^12^ The interaction between these surfaces and water has been a focus of much of this work due to their unique photochemical properties.^13,14^ From the LT perspective, their usefulness comes from their unique acid/base properties as a result of their negative surfaces, affording substantial capacity for cation exchange.^7^

Recently, a LT material, with a structure similar to those in literature,^15^ was discovered by Badr and coworkers.^16,17^ This discovery produced a one-dimensional (1D), by the quantum-mechanical defnition, LT phase,henceforth referred to as one-dimensional lepidocrocite titanate nanofilaments (1DLs). The zigzag structure of edge-sharing TiO_6_ octahedra shown in Figure 1A is unmistakably that of LTs, something visible in high-resolution transmission electron microscope micrographs.^18^ What distinguishes 1DLs from the LTs outlined in the literature is their width in the *c-*direction (Figure 1B,C), which is a result of their synthesis conditions, discussed later. When dried, unless arrested via polymer wrapping,^19^ 1DLs bond in the *c*-direction to form ≈3 to 5 nm assemblies.^18^ Heavily diluting the colloidal suspension can also assist in retarding this assembly; this can also be used to tune the band gap of the resulting filtered films.^20^ In most cases, the band gap energy is ≈4.0 eV, a result of the quantum-rod nature of 1DLs.^21^ This assembly can also be controlled by modifying the solvent system of the suspension, giving the final product a variety of different morphologies.^22^ In the LT literature, the narrowest width reported in this direction is ≈10 nm,^23^ achieved through complex top-down processing. In many other studies, the products are much wider, which, in some cases, results in tubular morphologies as a result of scrolling.^3,4,6^

**Figure 1:**
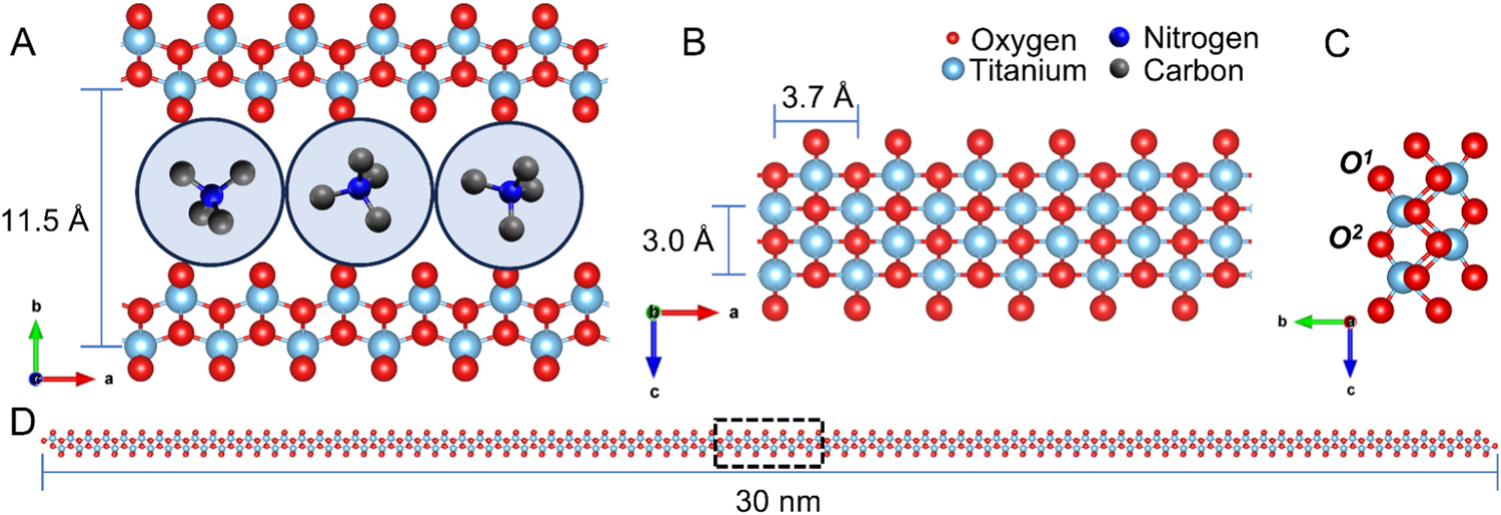
Structure of 1DLs. (A) *a-b* plane, (B) *a–c* plane, and (C) *b-c* plane of the 1DL base unit. Stacking shown in (A) is ABA stacking with TMA^+^ between the layers, giving a *d*-spacing (*b*/2-parameter) of 11.5 Å. Blue circle corresponds to hydration shell of TMA^+^ from molecular dynamics calculations.^37^ *a-* and *c-*parameters are shown in (B) and are 3.7 and 3.0 Å, respectively. (D) *a-b* plane of a single 1DL filament, dashed box corresponds to area shown in (A), showcasing its polymer-like aspect ratio.

Synthesizing 1DL is remarkably simple, employing many Ti-based precursors (*i.e*., carbides, borides, nitrides, oxysulfate, etc.),^16,24^ with tetramethylammonium hydroxide (TMAOH) at ambient pressures, temperatures <100 °C, in polyethylene bottles. During this reaction, the 1DLs grow along the *a*-direction (Figure 1A), while being restricted by the TMAOH^25^ from growing in the *b-* and *c-*directions (Figure 1B,C, respectively). 1DLs generally exist as ≈30 nm long snippets (Figure 1D).^26^ When alkali hydroxides are used instead of TMAOH, phases similar to the alkali LTs reported in the literature^3,4,9,27^ are obtained.^28^ 1DLs have demonstrated efficient ion exchangeability for organic and inorganic cations and even sensitization effects by common textile dyes.^29–32^ They have also been shown to be effective photocatalysts, particularly after thermal treatment.^33,34^ Additionally, they have been investigated for use as a sulfur host in lithium–sulfur batteries, among other applications.^16,35,36^

Herein, we explore the 1DL washing procedure, colloidal fabrication, and stability from a zeta (ζ) potential perspective. We also present the stability of dried 1DL solids, in terms of resuspension and dissolution under acidic conditions, measured by an inductively coupled plasma triple quadrupole mass spectrometer (ICP-QQQ). We discuss the Fourier transform infrared spectrometer (FTIR) spectra of dried 1DLs, pre- and post-acid treatment.

In our recent work, we discovered that 1DLs form highly compressible hydrogel networks through hydronium cross-linking^38^ and soft solvogels through solvent exchange.^22^ The work presented herein follows up on these studies to further the understanding of the surfaces of these unique materials that can arguably be described as inorganic polymers. In this sense, the 1DL surfaces were characterized from an acid/base perspective. It is apparent that 1DLs offer a fundamental negative charge of ≈1.8 mmol/g, equivalent to their ion exchange capacity (IEC), measured via multiple methods, which will be summarized in the following. In the colloidal state, it is possible to acidify 1DLs further, giving a maximum hydronium (H_3_O^+^) uptake of ≈4 mmol/g before the 1DLs become unstable. Using this information, various cation-cross-linked hydrogels could be obtained from various cations, including Li^+^, Mg^2+^, and Fe^3+^, among others. To study this gel state, the rheological properties of 1DL/mineral acid (HCl) cross-linked gels were measured and the “gel-state” viscosity was quantified for the first time. The requirements for gelation are simply to have a high concentration of 1DL colloid (>10 g/L) and a cation with sufficient charge density to cause a change in the interlayer chemical environment that leads to water displacement. When less water is displaced (Li^+^), a soft hydrated gel is formed; in contradistinction, when a larger cation, with a higher charge, *e.g*., Ba^2+^, is used, hard gels that displace substantial water are formed instead.

## Experimental Section

### Materials

The materials utilized in this study were titanium diboride, TiB_2_, (as received, 99.9%, 325 mesh; Thermo Fisher Scientific, Waltham, MA), tetramethylammonium hydroxide, TMAOH (as received, 25% [w/w] aqueous 99.9999%; Alfa Aesar, Ward Hill, MA), ethanol, EtOH (200 proof; Decon Laboratories, King of Prussia, PA), hydrochloric acid, HCl (as received, trace metal grade; Fisher Chemical, Pittsburgh, PA); lithium chloride, LiCl, sodium chloride, NaCl, potassium chloride, KCl, magnesium chloride, MgCl_2_ (as received, >99%, Thermo Scientific Chemicals, Waltham, MA), calcium chloride, CaCl_2_, and magnesium chloride, MgCl_2_ (as received, >99%, Thermo Scientific Chemicals, Waltham, MA), and iron­(III) chloride, FeCl_3_, (as received, >98%, Thermo Scientific Chemicals, Waltham, MA), all anhydrous, and barium chloride dihydrate, BaCl_2_·2H_2_O (as received, >99%, Thermo Scientific Chemicals, Waltham, MA). Ultrapure deionized water, <18.2 mΩ/cm, was utilized for all methods outlined below.

### 1DL Fabrication

Ten grams of TiB_2_ powder was added to 87.5 g of 25 wt % TMAOH aqueous solution in a 250 mL high-density polyethylene (HDPE) bottle vented with a single 23-gauge needle (Warning: The reaction can produce significant amounts of H_2_ gas and should ***not*** be carried out in ***closed systems***. All work should be carried out in fume hoods while mitigating pressure buildup.) The bottle was heated and shaken in an incubator (Labnet International Shaking Incubator, NJ) at 200 rpm and 80 °C for 4 d. The resulting sediment was combined with EtOH, vortex shaken and centrifuged at 3500 rpm for 2 min. The clear supernatant was discarded after each wash. This was repeated until a pH ∼7 was achieved (usually 3 times), measured using pH strips.

To form 1DL porous mesoscale particles (PMPs), the washed solid was dried at 80 °C overnight and crushed in a mortar and pestle. Importantly, to form PMPs, it is crucial *not* to introduce water prior to drying.

To form 1DL colloidal suspensions, water was added to the EtOH-washed product, and the material was suspended by vortex mixing. The mixture was centrifuged at 5000 rpm for 1 h, resulting in a colloidal suspension while any unreacted powders settled to the bottom. At this stage, the concentration of the suspension is usually ≈40 g/L. This was estimated by vacuum filtering 2 mL of the suspension through a 25 μm thick microporous monolayer polypropylene membrane (Celgard 3501, Celgard, NC) over a fritted glass filter apparatus. Once filtered, the solid was dried in an oven, under vacuum, at 80 °C overnight, and the weight of the residue was measured. The suspensions were then diluted with ultrapure water to the required concentrations.

### Thermogravimetric Analysis (TGA)

TGA scans were conducted using a thermal analyzer (PerkinElmer TGA 7 Series) with a heating rate of 20 °C min^–1^ under a nitrogen atmosphere, spanning the temperaure range from 50 to 600 °C.

### 1DL Acid Stability via ICP-QQQ

Ten milligram portion of finely crushed 1DL powders - both dry filtered films and PMPs - in 10 mL of acidified water was shaken for 24 h at room temperature/20 °C at 200 rpm. The pH of each solution is listed in Table S1. A fully dissolved film was prepared by dissolving 10 mg of 1DL film in 10 mL of aqueous 12 M HCl at 80 °C. An aliquot of 1 mL from each was taken. If possible, the samples were filtered through *a* < 0.45 μm PTFE syringe filter. All aliquots (regardless of whether they were filtered or not) were then diluted to 50 mL with ultrapure water (*i.e*., a 50× dilution). Each sample was introduced into an ICP-QQQ (8900 with SPS 4 autosampler, Agilent, Santa Clara, CA). The samples were run in helium mode at mass 47 using ultrapure water as the blank, using a Ti calibration standard (5190–8545, Agilent Technologies, Santa Clara) with a Sc internal standard to determine their concentration.

### Acid Exchange Process, FTIR, and XRD Analysis

The acid-exchanged powders were produced by mixing 100 mg of finely crushed 1DL filtered film or PMP powders with 45 mL of aqueous 10 mM HCl. The mixtures were then bath sonicated for 10 min and filtered over the same filter setup as above. The resulting solid was rinsed with 50 mL of water to wash away residual TMA^+^ in the filtration setup. The solid was allowed to filter fully before drying at 80 °C under vacuum for 2 h.

FTIR spectra of resulting powders were obtained at a resolution of 4 cm^–1^ in the 400 to 4000 cm^–1^ range (INVENIO-R with ATR attachment, Bruker Corp., Billerica, MA). The powders were placed directly on the ATR crystal, and pressure was applied by twisting down the hammer. The raw spectra were corrected by using a standard ATR correction in the Opus software.

X-ray diffraction (XRD) patterns were acquired with a diffractometer (MiniFlex 600 benchtop XRD, Rigaku, Tokyo, Japan) equipped with a Cu–K_α_ radiation source, scanned from 5 to 65° 2θ with step increments of 0.02 s^–1^ and a 1 s hold time.

### ζ-Potential

Electrophoretic mobility measurements were carried out in a DTS-1070 folded capillary cell supplied by Malvern Panalytical, utilizing a Malvern Panalytical Zetasizer Nano ZS instrument with a 633 nm red laser. Samples of the desired concentration and pH were flushed through the folded capillary cell, and the measurement was carried out on the third filling. Measurements were only carried out if there was good wetting/contact and if there were no inclusions or air bubbles in the cell. Measurements were performed at a set interval of temperatures, each with three total replications, each containing 11 runs. Before each measurement, samples were left to equilibrate at their desired temperatures for 90 s. The ζ-potential was then calculated using Smoluchowski’s approximation.

### Acid Response of 1DL Colloids

The pH of 1DL suspensions with 1 and 0.1 g/L concentrations, with an initial volume of 200 mL and pH 11.25 (increased using 0.1 M TMAOH), was measured as a function of the volume of 0.1 M HCl added. The HCl was added dropwise to the suspensions using a micropipette. pH readings were taken using a pH probe that was allowed to stabilize prior to the measurements. As the pH decreased, the time for automatic stabilization increased drastically (up to 10 min per addition).

To quantify the equilibrium H_3_O^+^ adsorption, a solution of 1 mM HCl (actual pH 2.43) was added to 1DL suspensions (0.1 g/L, initial pH 10.4), according to Table S2. The mixtures were allowed to shake for 1 h at room temperature before measuring the pH. At high pH, a suspension was maintained. At pH <6, the suspension destabilized.

### Fabrication of Gel-like Solids

Unless otherwise noted, solutions of the cation–chlorides (HCl, LiCl, NaCl, KCl, MgCl_2_, CaCl_2_, BaCl_2_, and FeCl_3_) were mixed with water to a concentration of 0.1 M. A suspension of 10 g/L of 1DL was obtained. Mixtures (5 mL of 10 g/L 1DL and 0.85 mL of 0.1 M cation–chloride) were mixed via vortex shaking and allowed to sit undisturbed for 15 min before inverting them for imaging. Mixtures of 5 mL of 10 g/L 1DL and various volumes of 0.1 M cation–chlorides were combined via vortex shaking and allowed to sit undisturbed for 1 week before being inverted for imaging. A mixture of 5 mL of 10 g/L 1DL and 0.068 mL of 5 M LiCl was combined via vortex shaking and allowed to sit undisturbed for 15 min before being inverted for imaging. A mixture of 5 mL of 10 g/L 1DL and 0.17 mL of 0.5 M NaCl was combined via vortex shaking and allowed to sit undisturbed for 15 min before being inverted for imaging.

See Table S3 for a summary of the preparation of all samples.

### Rheological Measurements

The rheological properties of the 1DL dispersions and gels were measured by using a rheometer (TA Instruments Discovery HR-3) with a 20 mm parallel plate at room temperature and a 1.0 mm gap distance in triplicate. The linear viscoelastic region (LVER) of the most viscous sample was determined to be 4% by measuring a 5% drop in storage modulus from the average of the plateau. 1% was chosen as the oscillation strain for all frequency sweeps.

## Results and Discussion

### Washing and Colloidal Fabrication

The fabrication process of the 1DL colloidal suspensions is surprisingly simple. After the reaction between TMAOH and the Ti-containing precursor, in this case TiB_2_, a slurry is formed (Figure S1A), comprised of a gray solid 1DL product and a highly basic TMAOH aqueous solution and borate byproducts. This slurry is *not* colloidally stable and can be easily separated by centrifugation (Figure S1B). At this point, to neutralize the mixture, and remove excess TMA^+^, a solvent is added as part of a solid/liquid extraction, or leaching, process. Since the discovery of 1DLs,^16,17^ EtOH was the solvent of choice. Adding water to the system prior to EtOH washing produces a highly basic colloidal suspension rich in excess TMA^+^.

Colloidal fabrication is reliant on the addition of water to the *wet* product—after neutralization with EtOH—producing a dark gray suspension (Figure S1C). If the product is dried, PMPs (Figure S2) are formed instead,^39^ and they *do not resuspend* in water (see ICP results below). It is postulated that this is due to the self-assembly of the 1DLs into two-dimensional (2D) lepidocrocite sheets during drying,^20,22^ as referenced by the PMPs^33^ having optical properties similar to those of delaminated 2D lepidocrocite titanates.^40,41^ During water addition to the wet EtOH-neutralized product, the pH of the system spikes from slightly acidic (*i.e*., pH ≈6, that of EtOH) to ≈10. It follows that the 1DL surface acts as a strong Brønsted–Lowry base, accepting protons from the water. These protons can be potentially localized on bridging oxygens (***O*** ^***2***^ in Figure 1C), like the TMA^+^, or the terminal oxygens (***O*** ^**1**^ in Figure 1C) on the 1DL surface.

To better understand how the 1DLs interact with various solvents, the following were used to wash the as-synthesized 1DLs: methanol, EtOH, propanol, isopropanol, butanol, and tert-butanol. TGA results are shown for RT dried samples in Figure S3A. In some cases, there is substantial residual liquid in the samples after drying at RT. Interestingly, the mass loss due to the decomposition of the structurally bound TMA^+^ is constant regardless of the solvent used to wash the 1DLs (Figure S3B). The various morphologies obtained were fully dried under vacuum and imaged in a scanning electron microscope (SEM) (Figures S4–S9). These results are discussed in further detail in SI Section S1 and will not be referred to again. In summary, 200 proof EtOH is still the most effective solvent for 1DL washing. This conclusion is based on its miscibility with water, low vapor pressure, and ability to extract TMA^+^ and OH^–^ from the mixture.

### Resuspension and Dissolution of 1DL Solids

Investigating the response/stability of 1DL solids under acidic conditions is important for many applications, especially aqueous catalysis and adsorption. We previously showed that PMPs are stable to acid to pH 2 and do not resuspend in water after they are dried, rendering them useful as sorbents of U and Th complexes, for example.^31,32^ Here, we study the stabilities of both finely crushed 1DL films and PMPs under acidic conditions.

When the dry, finely crushed, 1DL films are added to the acidic solutions, they resuspend across the pH 4–6 range (Figure 2, blue and red X). Resuspension in this case means that the liquid contains particles that cannot be filtered through *a* < 0.45 μm syringe filter (Figure S10), consistent with 1DL suspensions prior to drying. This resistance to filtering is most likely due to the greater viscosity of the 1DL suspension, as opposed to water or water containing dissolved Ti. The viscosity of 1DL suspensions is 2 orders of magnitude greater than water and will be discussed in further detail later. At pH 2 and 3, the liquid can be filtered, meaning there is no resuspension, thus any of the paritcles remain as particles or are dissolved. Here again, when analyzed via ICP-QQQ, there is no substantial dissolved Ti (Figure 2). These values (≈11 ppm) are <0.2% of the fully dissolved 1DL film (≈400 ppm—orange line, Figure 2). Said otherwise, the vast majority of the Ti atoms remain in solid form and are filtered out. Finally, at pH 1, PMPs and both films are slightly soluble in the solution (Figure 2); in all cases, the dissolution is <25% of their maximum dissolution concentration of Ti (orange line, Figure 2). The ICP-QQQ results for the 1DL PMPs shown in Figure 2 (dark gray) support the work by Wang et al.^31^

**Figure 2:**
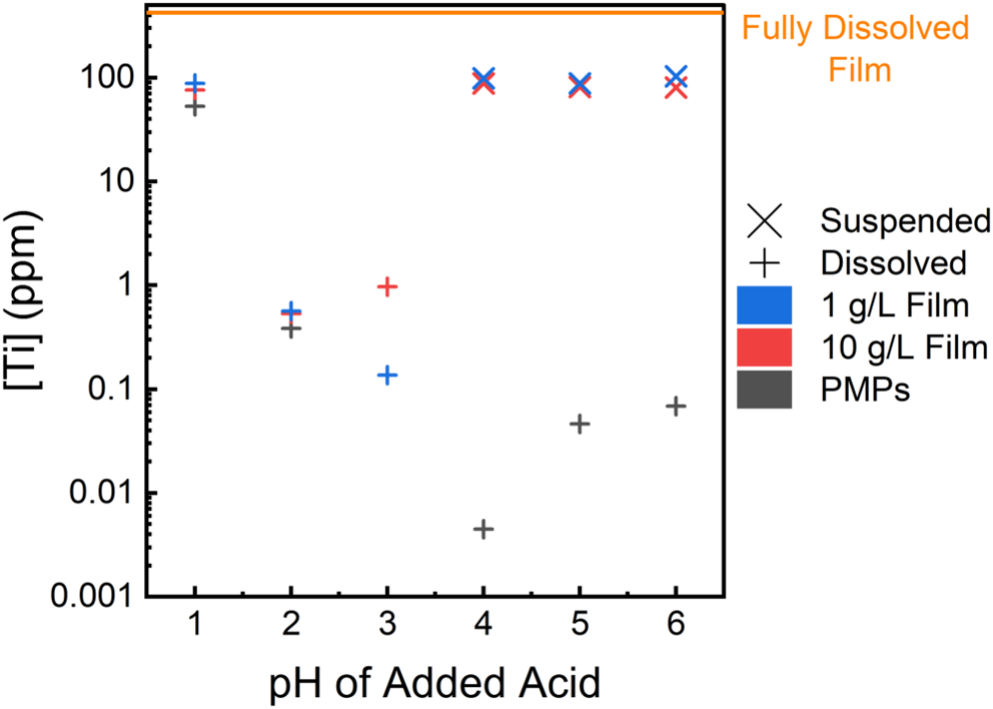
Semilog plot of Ti concentration in liquid state measured using ICP-QQQ after treating 1DL films and PMPs with water at various acidic pH values. X corresponds to resuspended, and + corresponds to dissolved. Samples were prepared by shaking 10 mg of solid in 10 mL of acidified water with pH values shown for 24 h at RT at 200 rpm in an orbital shaker. Ti concentration of a fully dissolved film, depicted by the orange line, was obtained by dissolving 10 mg of 1DL film in 10 mL of 12 M HCl at 80 °C for 1 h. pH values are rounded; actual values are listed in Table S1. Results and labels are color coordinated.

To summarize this section, the most effective pH to H_3_O^+^ exchange 1DLs is between 2 and 3, where dissolution and/or resuspension of the solid is quite small (Figure 2). This is useful for producing H_3_O^+^ exchanged products while avoiding dissolution.

Interstingly, after acid exchanging the 1DLs, there is a substantial volume increase when wetted by the solution for both the PMPs and films (Figure S11A,C), which is lost when dried again (Figure S11B,D). The PMP powders undergo a substantial color change from gray (Figure S2A) to black (Figure S11A), while the films remain gray (Figure S11C). The films show a *d*-spacing shift of the low-angle X-ray diffraction (XRD) peak from ≈11.5 Å (2θ = 7.7°) to 9.5 Å (2θ = 9.3°) (Figure S12), consistent with earlier work on 1DL PMPs, resulting from exchanging the TMA^+^ with H_3_O^+^.^39^

These results also suggest that the 1DL films can be resuspended, most probably due to their increased level of hydration, while the PMPs *do not* resuspend at any pH. This work also establishes a baseline for 1DL stability in high-acid conditions.

### Solids Characterization and Hydronium Exchange

FTIR is useful in understanding the organic component (TMA^+^) and H_2_O/OH environments within the 1DL system. The FTIR spectra obtained on a finely crushed 1DL film, PMPs and hydronium-exchanged films are shown in Figure 3A–C, respectively. A summary of the peak positions and their assignments is listed in Table 1. An analysis of these results makes it clear that the 1DL films have a FTIR spectrum slightly different from that of their PMP counterparts (compare Figure 3A,B). Comparing the relative intensity between the OH^–^ region peaks in the 3300–3400 cm^–1^ range and the peak at 1670 cm^–1^ (black solid, Figure 3) to the Ti–O backbone peaks in the 400–900 cm^–1^ range (blue dotted, Figure 3),^42,43^ it is reasonable to conclude that the films contain more water. This is not too surprising since PMPs do not encounter water prior to drying; the amount of OH in their system results from the synthesis process or atmospheric water. Additionally, there is a shift in the relative intensities between the 3320 and 3400 cm^–1^ peaks, which is indicative that the films have a higher concentration of free OH, as opposed to OH that is participating in H-bonding.^44^ In both systems, TMA^+^ peaks (red dashed lines, Figure 3) are apparent.^45^

**Figure 3:**
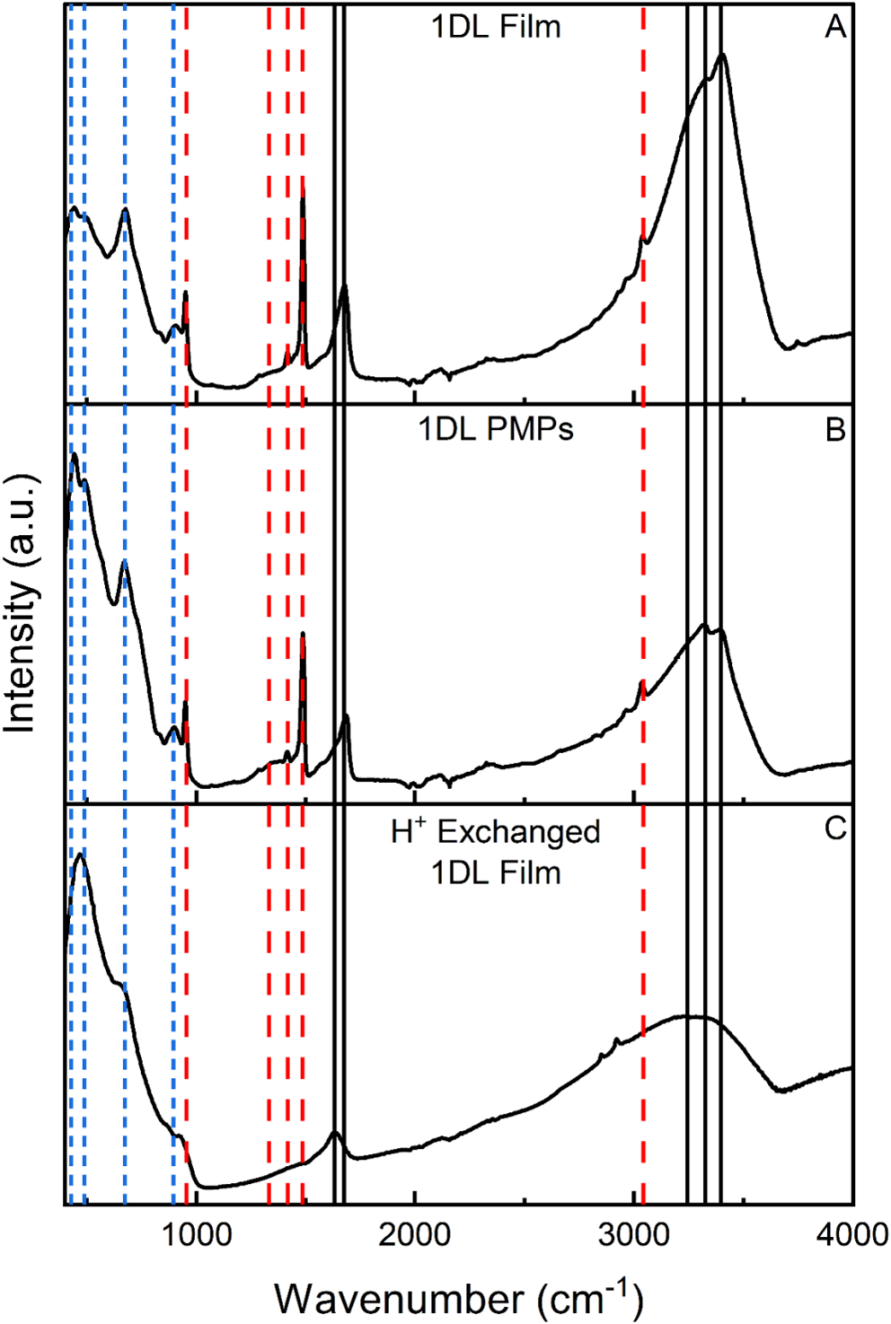
Representative FTIR spectra of 1DLs. (A) Vacuum filtered film, (B) PMPs, and (C) H_3_O^+^-exchanged film. Vertical lines are associated with modes assigned in Table 1: (Blue dotted) Ti–O backbone, (Red Dashed) TMA^+^, and (Black Solid) H_2_O/OH.

**Table 1:** FTIR Peak Assignments for Spectra Shown in Figure 3,^a^

wavenumber (cm^–1^)	type	assignment	comments
440	medium	Ti–O backbone	all samples
500	sharp	Ti–O backbone	all samples
680	medium	Ti–O backbone	all samples
904	medium	Ti–O backbone	all samples
950	sharp	TMA^+^	pre-exchange (A, B)
1250–1400	shoulder	TMA^+^	pre-exchange (A, B)
1415	sharp	TMA^+^	pre-exchange (A, B)
1490	sharp	TMA^+^	pre-exchange (A, B)
1635	medium	H_2_O/OH	postexchange (C)
1670	sharp	H_2_O/OH	pre-exchange (A, B)
3040	sharp	TMA^+^	pre-exchange (A, B)
3250	broad	H_2_O/OH	postexchange (C)
3320	sharp	H_2_O/OH	pre-exchange (A, B)
			higher in PMPs (B)
3400	sharp	H_2_O/OH	pre-exchange (A, B)
			higher in film (A)

a (A–C) in Column 4 are callouts for panels in Figure 3.

When the TMA^+^ is replaced with H_3_O^+^ using HCl in the films, there are drastic changes in the FTIR spectra (compare Figure 3C to A or B). The most obvious change is the loss of all TMA^+^ peaks (red dashed lines, Figure 3). Additionally, there is a red shift and broadening in the OH regions (black solid lines, Figure 3)—1670 to 1635 cm^–1^ and 3320/3400 to 3250 cm^–1^—signifying an increase in H-bonding between the OH groups on the surface and adsorbed water, respectively.^44^ Although FTIR is not generally used to study the Ti–O bond, these regions (blue dotted lines in Figure 3) are relatively unchanged. The XRD patterns in Figure S12 also suggest no major change in the Ti–O backbone region (above the peak at 2θ ≈ 48°). Additionally, the spectrum shown in Figure 3C is quite similar to the FTIR spectrum of 2D lepidocrocite sheets pioneered by Sasaki and co-workers.^7^

Note that the presence of sharp peaks at 3320 and 3400 cm^–1^ in the 1DL samples (black solid lines, Figure 3A,B) are most probably due to adsorbed water on the surface of 1DLs and TMA^+^ hydration shells based on the uniqueness of this feature to both 1DL films and PMPs, pre-H_3_O^+^ intercalation. Future theoretical work is planned to elucidate this characteristic of the 1DL spectra.

### Colloidal Stability and pH Response of 1DL Suspensions

To obtain a point of zero charge (PZC) for 1DL suspensions, a ζ-potential study as a function of pH was conducted (Figure 4A). The ζ-potential at room temperature of a 1 g/L 1DL aqueous suspension was ≈−85 mV at its unadjusted pH of ≈10.4. Decreasing the pH leads to a reduction in the magnitude of the ζ-potential (−65 mV at pH ≈7) (Figure 4A). However, at a pH of 6 or less, the 1DLs agglomerate into gel-like globules (Figure S13) that cannot be introduced into the folded capillary cell required for ζ-potential measurements. Further experimentation into the pH response of the ζ-potential of 1DL colloids will be the subject of future work.

**Figure 4:**
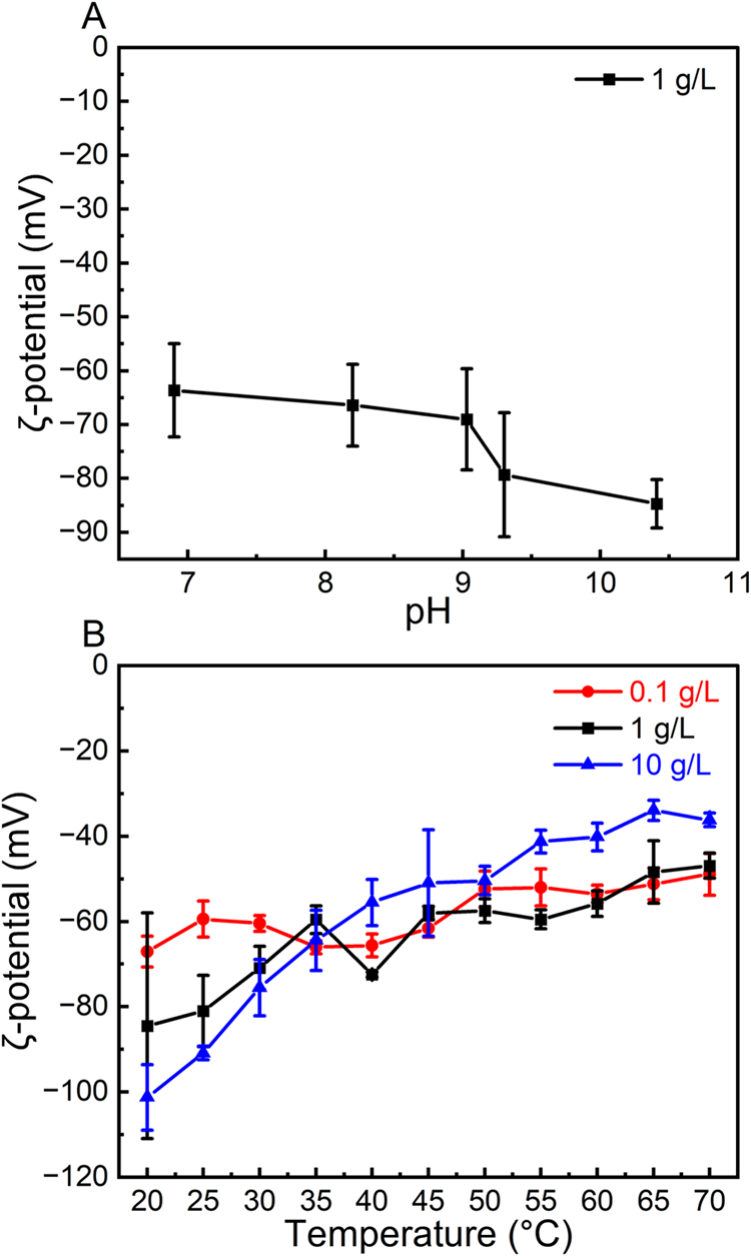
Average ζ-potentials of 1DL of different concentrations. (A) ζ-potential as a function of pH for 1 g/L 1DL at 20 °C. (B) ζ-potential as a function of temperature of 1DL suspensions at concentrations indicated on the panel at their unadjusted pH ≈10. Error bars show standard deviation across 3 replicates. Results and labels are color coordinated.

To understand the effects of concentration and temperature on the ζ-potentials of 1DL suspensions at their unadjusted pH are shown in Figure 4B. Interestingly, the more concentrated the 1DL colloid, the greater the magnitude of the ζ-potential at RT, *i.e*., ≈ =100, 85, and 70 mV for 10 g/L (blue), 1 g/L (black), and 0.1 g/L (red), respectively (Figure 4B). This is most likely due to small variations in pH between samples, where the more concentrated samples are more basic. However, the lower the colloidal concentrations, the lesser the reduction in magnitude in ζ-potential as the temperature increases (red, Figure 4B). It is possible that the differences in viscosity between the dilute (close to water) and concentrated 1DL suspensions is impacting the results due to the viscosity correction for ζ-potential measurements. There is also the potential that the loss in stability is due to a temperature-induced disruption of the local hydrogen bonding environment between the colloidal 1DLs, leading to adjacent filaments agglomerating through bonding along the *c*-direction. In summary, the 1DL suspensions, with concentrations in the 0.1–10 g/L range, remain quite stable across the 20 to 70 °C temperature range.

Following up on the unique gelation response of 1DL suspensions to acid,^38^ their acid/base properties were further studied. 1DLs are strong proton sinks at both 0.1 g/L (blue, Figure 5A) and 1 g/L (red, Figure 5A). The 10 g/L suspension was omitted due to its tendency to form a gel-like solid (Figure S14), which will be discussed in further detail later.

**Figure 5:**
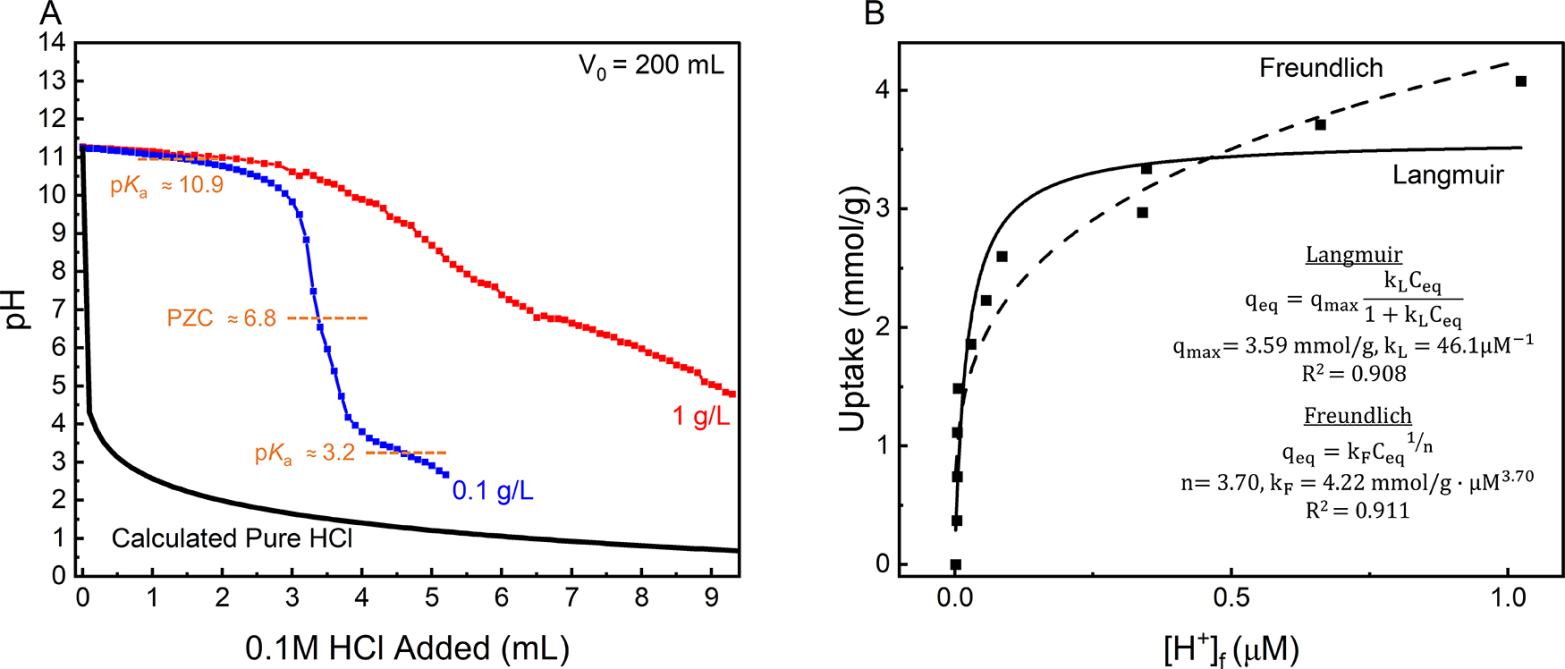
Acid response of 1DL colloidal suspensions. (A) The titration curve of 1DL suspensions with concentrations of 1 g/L (red) and 0.1 g/L (blue). The black line shows the calculated pH if acid is added directly to the solution *without* 1DL. pH readings were taken using a pH probe and allowed to stabilize prior to noting their values. Initial volume was 200 mL. Orange dashed lines note the p*K*_a_ values and PZC of 1DLs. (B) Equilibrium adsorption of hydronium ion from solution over 1DL colloidal suspensions. Samples were prepared according to Table S2 and allowed to shake for 1 h at RT before pH was measured. Inset shows Langmuir and Freundlich isotherm fittings of the experimental data. Initial 1DL concentration of 0.1 g/L at a total volume of 10 mL. Results and labels are color coordinated.

The 0.1 g/L suspension (blue, Figure 5A) shows a substantial drop in pH after being acidified below pH ≈10, and then begins to level out at pH 4. At 1 g/L, the 1DLs uptake a substantial number of protons and begin to agglomerate when the pH is reduced to 6, which is why the titration stopped at this point. Here, the 1DLs begin to self-buffer, making it difficult to obtain stable and repeatable pH readings in a reasonable amount of time. Because of this, and to reduce the potential impact of the suspension effect, the batchwise proton adsorption study was only carried out on the 0.1 g/L suspension.

Figure 5B illustrates the equilibrium adsorption of protons over a 0.1 g/L suspension. This plot *only* includes results to pH ≈6 (1 μM). When 1DLs were acidified further, they began to self-buffer. Interestingly, this response was absent in the stepwise titration (blue, Figure 5A), which was carried out at a higher volume (200 vs 10 mL).

The equilibrium pH values as a function of the addition of HCl are shown in Figure S15 and listed in Table S2. Below a pH of 6, the suspensions were unstable. Furthermore, the pH results became quite noisy and needed a long time to equilibrate. Interestingly, below pH 3, the 1DLs apparently release protons (Figure S15), which could be the result of a phase change from lepidocrocite to anatase—seen in both our work^38^ and in the literature on protonic layered titanates.^46^

The results shown in Figure 5B were fit with both the Langmuir and Freundlich isotherms. The latter gave a better fit (Figure 5B). This is reasonable, assuming that the heterogeneity of the 1DL surface stems from their ability to uptake protons via two possible mechanisms: direct protonation of terminal oxygens labeled ***O*** ^**1**^ in Figure 1C and/or ion exchange in the interlayers at the bridging oxygens labeled ***O*** ^***2***^ in Figure 1C.

Understanding the 1DL surface is best discussed through the concepts of surface acidity. The ***O*** ^**1**^ atoms can exist in three forms: –O^–^ at high pH, −OH at the PZC, and −OH_2_^+^ at low pH. In reality, and depending on the solution’s pH, some mixture of the three probably exists, consistent with the TiO_2_ literature.^47^ Exploring the proton adsorption energies of ***O*** ^**1**^ as opposed to ion exchange in 1DLs is the subject of ongoing theoretical work. However, one can theorize that primary protonation is the most energetically favorable, followed by the exchange of TMA^+^ with hydronium and finally the dual protonation of the surface.

These equilibria can be noted as p*K*_a_, where there are two values for 1DL, the former being at ≈10.9 and the latter being at ≈3.2, which fit well with the theoretical work on the p*K*_a_ of various TiO_2_ surfaces.^48^ When fabricating 1DL suspensions, the addition of water to the damp EtOH-washed PMPs leads to the formation of a suspension with a pH of ≈10, which means 1DLs equilibrate near their higher p*K*_a_ during initial suspension.

A PZC value can now be extracted by averaging the two p*K*_a_ values, which gives 6.8, just slightly higher than the upper bound of anatase TiO_2_ in the literature.^47^ Using this titration method is useful because it is not reliant on ζ-potential measurements. That being said, it is unclear at this point why the PZC of 1DL suspensions reported here is higher than the 1DL PMPs shown in our earlier work.^31^ However, this value is reasonable based on the tendency of 1DL suspensions to destabilize and self-buffer around pH 6 (discussed earlier).

### Fundamental Charge of 1DLs

An important aspect of ion-exchangeable materials is their IEC, which is essentially a measure of their fundamental charge. IEC is generally measured by two methods: one by quantifying the counterions in as-synthesized materials and/or by quantifying the ionic uptake of an external counterion. In previous published work, we did not set out to measure the IEC of 1DLs directly. Instead, during our pilot study on H_3_O^+^-cross-linked 1DL gels, we quantified the TMA^+^ content in the as-synthesized 1DL colloids using nuclear magnetic resonance (NMR) on the postexchange supernatant.^38^ This value came to 13 wt %, that converts to ≈1.8 mmol TMA^+^ per g of 1DL. Similarly, the adsorption of singly charged cationic dyes—rhodamine 6G, crystal violet, and malachite green—all resulted in maximum uptakes, determined through the RT equilibrium Langmuir expression, between 1.8 and 2.0 mmol of dye per gram of 1DL.^29,30^ Using H_3_O^+^ to evaluate the IEC of 1DLs is difficult, in that 1DLs can both accept the H_3_O^+^ as interlayer cations, or as H^+^ that localize on the terminal O groups. Further work is ongoing in quantifying the uptake of elemental cations, including alkali, alkaline earth, and transition metals, by 1DLs to further cement this value.

To summarize, the near convergence of the cation displacement and dye uptake values implies that the IEC of 1DLs is at a minimum 1.8 mmol/g (meq/g). To put this value into perspective, Table 2 lists the IEC values for various ion-exchange materials, both nanomaterials and polymeric membranes. This illustrates the potential of 1DLs as next-generation ion-exchange materials.

**Table 2:** IEC Values for Various Ion-Exchange Materials

material	IEC (meq/g)	source(s)
1DL	1.8	^29,30,38^
Ti_3_C_2_ MXene	0.71	^49^
bentonite (Arizona)	1.2	^50^
bentonite (MX-80)	0.76	^50^
nafion	1	^51^

In terms of interlayer cations, the IEC is sufficient for understanding the 1DL charge. However, for a full picture of the 1DL surface, both the adsorption of interlayer hydronium cations and the adsorption of edge protons must be considered. The concentration of interlayer and edge sites can be calculated using the maximum uptake shown in Figure 5b (≈4.0 mmol/g), assuming that the ***O*** ^**1**^ groups are singly protonated, since the lower pH limit of the measurements was near the 1DL PZC (pH ≈6.8). With this new understanding that 1DLs offer an interlayer IEC of 1.8 mmol/g, the concentration of 2.2 mmol of ***O*** ^**1**^ sites per gram of 1DL can be calculated.

### Time Dependency of Cation-Cross-Linked Gelation

Throughout the literature, there is ambiguity in the usage of the term “gel”. We subscribe to the definitions by Malkin et al., by which the products mentioned throughout the remainder of this paper are more akin to gel-like solids, defined simply as the solid-like state of a yielding liquid.^52^

With a new understanding of the IEC of 1DLs, the formation of 1DL gel-like solids was studied systemically. In our pilot study on 1DL-H_3_O^+^ hydrogels, weak organic acids were utilized to deliver the specified amount of hydronium needed for gelation—while we had difficulty in controlling gelation using mineral acids (like HCl) without inciting a phase change from lepidocrocite to anatase/rutile (See Figure S5 in Ref. 38). In retrospect, and based on the IEC values discussed here, it is clear that we supplied nearly 30 times the amount of acid necessary to accomplish full cation exchange (50 mmol/g as opposed to 1.7 mmol/g). This excess probably dehydrated the 1DLs sufficiently to precipitate mixed-phase titania.^46^

The results of exchanging TMA^+^ with various cations and their propensity for gel formation are shown in Figure 6A–D. Table S3 gives the details of every run. All of the mixtures gelled within 2 d, but were allowed to sit for 1 week to establish that no further changes occurred. In this figure, red letters denote no gel formation; blue, hard gels and green, soft gels. Hard and soft gels are, respectively, defined as those that *reject* water and those that do not. Said otherwise, the volume of the hard gels is less than the initial volume. For the Ba^2+^ sample (0.85 in Figure 6B), the mass loss was ≈30%, calculated by weighing the displaced water.

**Figure 6:**
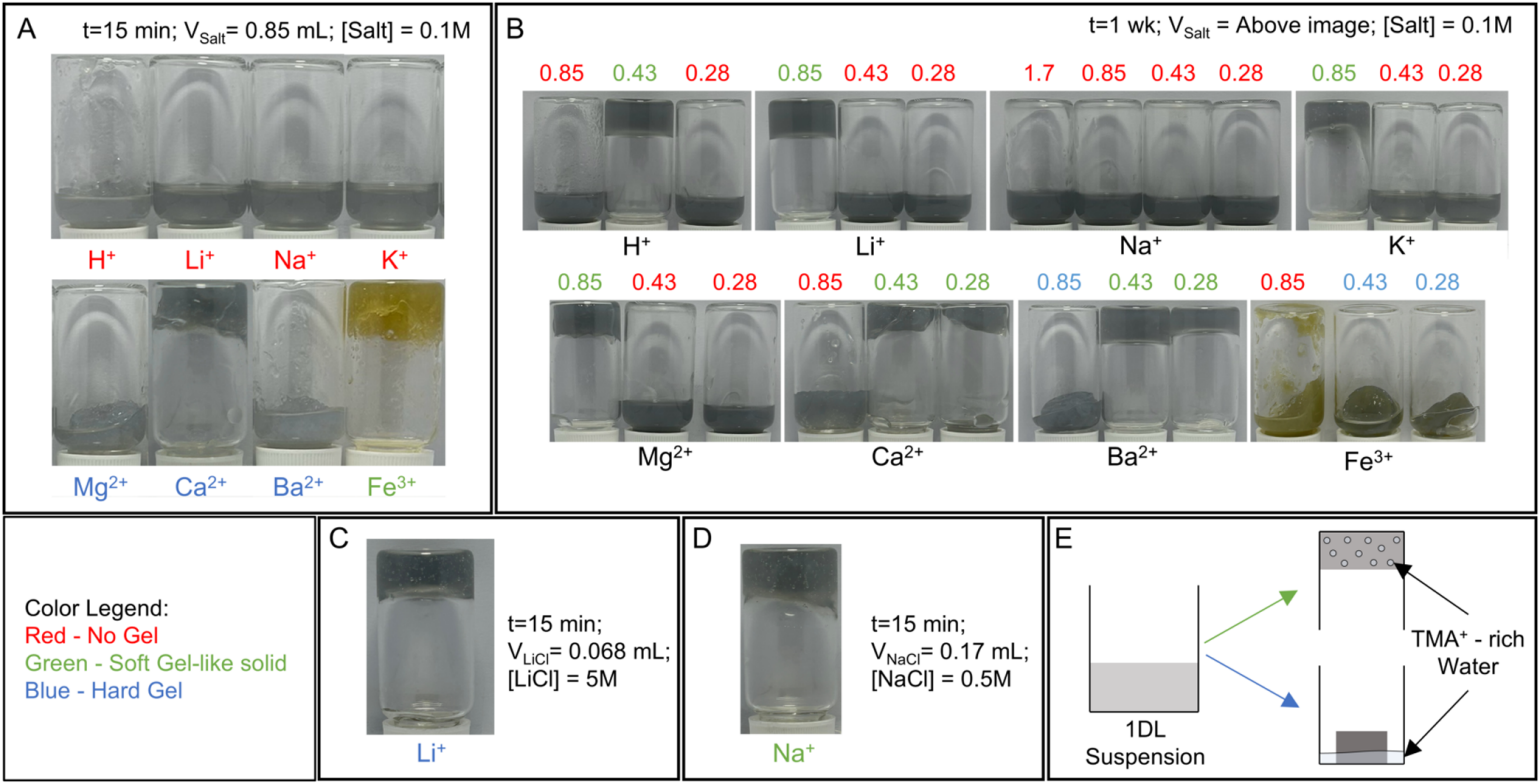
Photographs of TMA 1DL powders after addition of various cation–chloride mixtures. (A) Mixtures (5 mL of 10 g/L 1DL, and 0.85 mL of 0.1 M cation–chloride) were combined via vortex shaking and allowed to sit undisturbed for 15 min, then inverted for imaging. (B) Mixtures (5 mL of 10 g/L 1DL, and various volumes of 0.1 M cation–chloride solutions) were combined via vortex shaking and allowed to sit undisturbed for 1 week, then inverted for imaging. Numbers above pictures denote volume of salt added in mL. (C) Increased Li^+^ concentration to speed up the gelation process. (D) Increased Na^+^ concentration leads to a soft gel-like solid. (E) Schematic of proposed gelation mechanism, showing bubbles of TMA^+^-rich water in soft gel-like solids and displaced TMA^+^-rich water in hard gels. Note: Solutions that did not form gels are labeled red, those that formed soft gel-like solids are labeled green, and those that formed hard gels are labeled blue. Compositions and results are summarized in Table S3.

Soft gels generally stick to the bottom of the vial, even when inverted, and hold most of their water (green letters, Figure 6). Hard gels “displace” substantial water, generally separate from the wall of the vial, and detach once inverted (blue letters, Figure 6). Quantifying the “softness” of these gels and gel-like solids through rheology is the subject of future work outside the scope of this work.

Based on the totality of the results shown in Figure 6A–D and summarized in Table S3, the most important conclusion we can draw is that whether a hard or soft gel forms is a complicated function of water content, cationic charges, and time. If the gels are left to sit undisturbed in a closed system, free-standing gels form across all cations for at least one composition (Figure 6B). Na^+^ requires the lowest water content to gel (0.015 M Na^+^ and 9.67 g/L 1DL). After 15 min, no monatomic cations form gels at low salt concentration (0.015 M final concentration). The divalent ones form hard gels. Fe^3+^ forms a yellow soft gel. Monatomic cations do not form hard gels, unless at high concentrations (0.067 M final concentration in Li^+^). Except for Ba^2+^, the hard gels formed at short times with Mg^2+^ and Ca^2+^, which convert to a soft gel and no gel, respectively, after a week. To form time-stable hard gels, Ba^2+^ and Fe^3+^ are recommended. Once formed, the hard gels are stable and will not deteriorate in the closed vial for at least 1 week.

### Proposed Gelation Mechanism

When discussing a possible gelation mechanism, one cannot ignore the fact that at select cationic concentrations, the gels actually *displace* water, in striking contradistinction to the more common hydrophilic hydrogels in the literature that do not.^53^ Further distinguishing the soft hydrogels (green entries in Table S3) is the lack of displaced liquid. The harder gels that are more resistant to deformation displace water (blue entries Table S3). This is also consistent with the organic acid gels in our pilot study,^38^ which all displaced some liquid.

Based on the totality of our results to date, we propose that when the TMA^+^ cations with their large water of hydration shells are replaced by the cations explored herein (Figure 6), a local change in the chemical environment surrounding adjacent 1DLs occurs that increases interfilament interactions, producing a gel network. Making the reasonable assumption that the presence of excess water interferes with gel formation, it follows that the lower the hydration sphere of the ion (*e.g*., Ba^2+^), the more apt the system is to form free-standing and harder gels. This mechanism explains why the hardest gels we obtained to date are the result of pure organic acids being added to concentrated 1DL colloids (≈40 g/L),^38^ which introduces the smallest amount of water possible to the system. This proposed mechanism also explains the formation of the methanol-based solvogels encountered in a separate study.^22^ In that system, the water is extracted by methanol, while TMA^+^ remains the countercation in the system. Additionally, this mechanism suggests that the removal of water is causing gelation, as a result of the ion exchange, thus possibly explaining the delayed gelation in some systems (see Li^+^ and K^+^ in Figure 6B). Cation exchange in solids in general, and 1DLs in particular, is usually a fast process, which was demonstrated in our work on 1DLs, where equilibrium is typically reached within ≈10 min.^29–32^ This explains why, in the cases of high concentrations of mineral acid or pure organic acids, gelation occurs almost instantaneously.^38^

To support this hypothesis, we aimed to increase the gelation time of the Li^+^ gels and cause gelation in the Na^+^ system by modifying the concentration of cation–chloride. Through increasing the LiCl concentration from 0.1 to 5 M, gelation occurred within 15 min (Figure 6C). Although this sample has a higher total Li to 1DL ratio (6.8 mmol/g as opposed to 1.7 mmol/g), the volume of the salt solution for the lesser ratios was not sufficient to cause the whole suspension to gel. In general, small gels formed within the bulk (SI Video S2). Following this same mindset, a soft gel-like solid can be formed from Na^+^ (Figure 6D) by increasing the salt concentration to 0.5 M and reducing the added volume to 0.17 mL, while still keeping the 1.7 mmol/g ratio.

The situation for the soft gel-like solids is slightly different since there is little change in volume upon gelation. It follows that gelation is caused by a phase separation between gelled regions, the local TMACl and water-rich emulsion-like “domains” within the matrix (Figure 6E). These domains form presumably due to the preference for TMA^+^ and Cl^–^ to be in water-rich regions, while the 1DLs and added cations separate into water-poor regions. When agitated, whether it be shaking or shearing, the domains are mechanically disturbed, which forces them together, producing a biphasic product (SI Video S3 shows the 0.85 K^+^ soft gel-like solid after agitating by hand). For the hard gels, the destabilization of the 1DL colloid is much more rapid, forcing the TMACl-rich water out of the gel matrix (Figure 6E).

### Gel-like State Rheological Properties

As mentioned above, H_3_O^+^ cross-linked gels see an immediate increase in viscosity, but not the formation of a free-standing gel. To support this fact, the rheological properties of various mixtures of 1DL and HCl were studied (Figure 7). The 1DL colloid (black, Figure 7A) shows a shear thickening type behavior. As acid is added, the shear thickening behavior is slowly reduced until the gel-like state is reached (green, Figure 7A). At that point, the suspension is exclusively shear thinning. It shows that the gel-like state of the mixtures is quantifiable with an increase in viscosity of 4 orders of magnitude. The maximum viscosity obtained was the 1.7 mL sample (equivalent to 1.7 mmol/g of 1DL) (Figure 7B). The loss of viscosity above this value can be attributed to the increase in water within the system. The authors acknowledge the complexity of studying the rheological properties of nanomaterial dispersions and that parallel plate geometry is not the best for low-viscosity systems. The results in Figure 7 are meant to provide some quantification of the gel-state transition based on acid concentration; thus, further rheological studies are justified and are the subject of future work.

**Figure 7:**
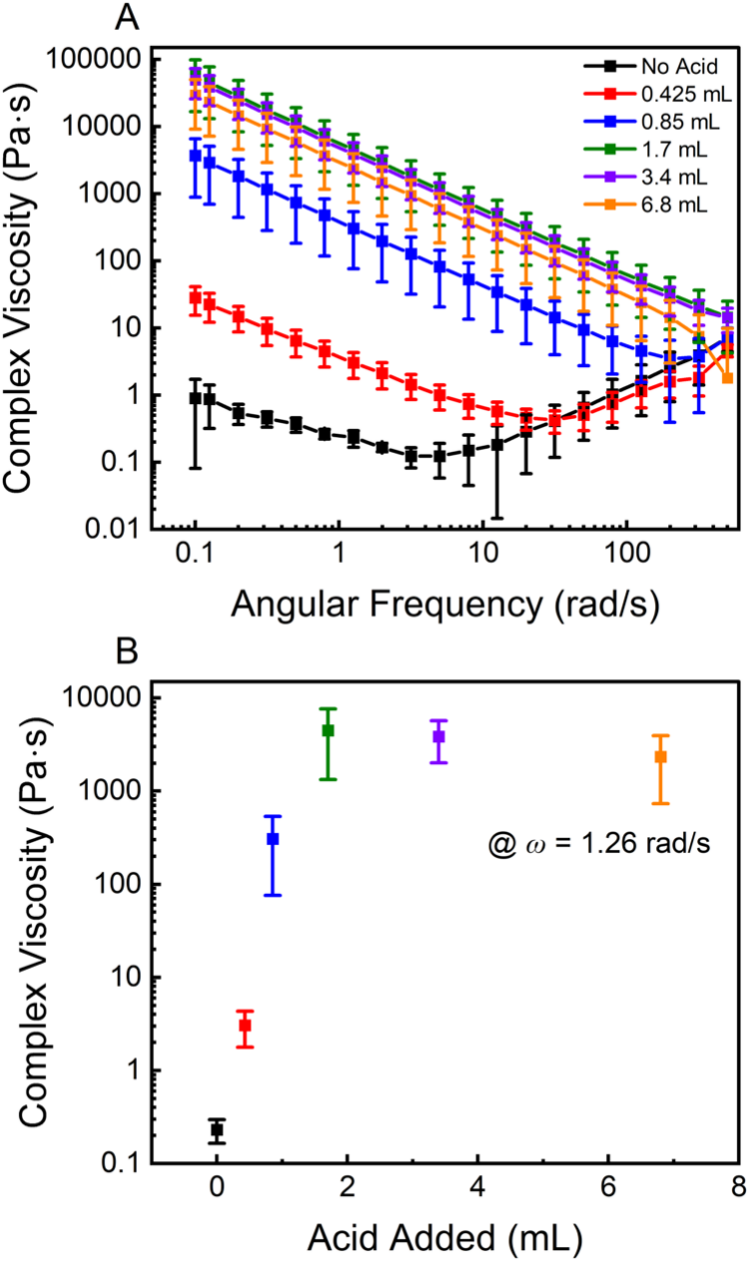
Rheological properties of 1DL H_3_O^+^-cross-linked gel-like solids. (A) Viscosity of each sample as a function of angular frequency (ω). (B) Complex viscosity as a function of volume of acid added, measured at *ω =* 1.26 rad/s. Samples were prepared by mixing a known volume of 0.1 M HCl solution with 10 mL of 10 g/L 1DL suspension and then running on a parallel plate rheometer with an oscillation strain of 1% and a 1 mm gap. Strain chosen from the LVER of the 1.7 sample (Figure S16). Results and labels are color coordinated. Error bars are the standard deviation of 3 replicates.

## Conclusions

Herein, we explored the surface properties of 1D quantum-confined lepidocrocite titanate materials. We show that 1DLs form stable aqueous colloidal suspensions up to temperatures of 70 °C. Furthermore, their unique acid/base properties and minimum IEC at 1.8 mequiv/g of 1DL (nearly double that of Nafion) suggest that they can be used as ion exchange materials/membranes. Further, as a result of their unique surface behavior, 1DLs form a gel-like solid simply through cation exchange and water displacement. This charateristic offers the first instance of controlled gelation through means other than acids.

In essence, 1DLs are *all* surface; thus, understanding their surface is core to future discoveries and advancements of this paradigm shift in nanomaterials.

## Supplementary Material









## Notes

Experimental information, including images, further discussion into and data for the TGA analysis, and additional results (PDF)
Solvent/1DL interactions (Video S1) (MP4)
Result of mixing 1DL colloid and concentrated LiCl (Video S2) (MP4)
Result of shaking the 1DL/KCl gel (Video S3) (MP4)
