# Supplementary material for: Surface Properties of Colloidal Quantum-Confined One-Dimensional Lepidocrocite Titanates: Insights into their Ion-Induced Gelation
Source: Langmuir. 2025 Sep 10;41(37):25176–86. doi: 10.1021/acs.langmuir.5c02076 (PMC12461908; doi:10.1021/acs.langmuir.5c02076)
Supplement: Supplementary file 1 [file la5c02076_si_001.pdf]

Surface Properties of Colloidal Quantum-Confined One-Dimensional Lepidocrocite Titanates: Insights into their Ion-induced Gelation

Adam D. Walter[a], Vanessa R. Morris[a], Jacob M. Nantz[a], Timothy F. Niper[b], Laura Galeano Tirado[b], Mary Qin Hassig[a], Abijah Gordon[a], Tongjie Zhang[a], Ahmed M. H. Ibrahim[a], Gregory R. Schwenk[a], Jairo A. Díaz A.[b], Andrew J. D. Magenau[a], Christopher Y. Li[a], Michel W. Barsoum[a,*]

[a]Department of Materials Science and Engineering, Drexel University, Philadelphia, PA, 19104, USA

[b]Department of Chemical Engineering, Rochester Institute of Technology, Rochester, NY, 14623, USA

*Corresponding Author (barsoumw@drexel.edu)

Tables

Table S1. pH values of acid solutions used in Figure 2. Related to the 1DL acid stability experiment. 4

Table S2. Summary of sample compositions used to obtain results shown in Figure 5B and Figure S15. In all cases, colloidal suspension volumes and total volume of mixtures were kept constant. Column 6 lists the final pH values. Last column describes the final product. 4

Table S3. Summary of the non-gelled, gel and gel-like solid 1DL products. Photographs of these products are shown in Figure 6. 5

Figures

Figure S1. 1DL wash procedure. (A) Slurry of TMAOH and 1DL resulting from the reaction. (B) Post-centrifugation of 1DL slurry shown in (A). (C) 1DL colloid after ethanol washing and suspension in water. 3

Figure S2. 1DL PMPs. (A) Photograph of 1DL PMP powder. (B) Low-mag SEM micrograph of 1DL PMPs. Inset shows particle size analysis. (C) High-mag SEM micrograph of a single PMP. (B,C) adapted from Badr et al. [1]. 3

Figure S3. TGA analysis of 1DL PMPs formed by washing with various solvents indicated on figure. Samples were dried at RT under vacuum prior to analysis. (A) TGA data for all solvents. Raw TGA data was normalized at 45 °C. (B) Relative mass loss from 250 °C to 400 °C for each solvent, corresponding to the region of structurally bound TMA^+. Curves are color coordinated to legend in (B). 7

Figure S4. Methanol-washed 1DL product. Photograph of RT dried product and SEM micrographs of the products dried at 100 °C at various magnifications. 8

Figure S5. Ethanol-washed 1DL product. Photograph of the RT dried product and SEM micrographs of the product dried at 100 °C at various magnifications. 9

Figure S6. Propanol-washed 1DL product. Photograph of the RT dried product and SEM micrographs of the product dried at 100 °C at various magnifications. 9
Figure S7. Isopropanol-washed 1DL product. Photograph of RT dried product and SEM micrographs of the product dried at 100 °C at various magnifications. Note residual liquid in the photograph. 10
Figure S8. Butanol-washed 1DL product. Photograph of RT dried product and SEM micrographs of product dried at 100 °C at various magnifications. Note residual liquid in photograph. 10
Figure S9. Tert-Butanol-washed 1DL product. Photograph of RT dried product and SEM micrographs of the product dried at 100 °C at various magnifications. Note residual liquid in photograph. 11
Figure S10. Re-suspended 1DL particles in water. Filter is a < 0.45 μm PTFE syringe filter. Re-suspended particles resist filtration through such filters. 12
Figure S11. Photographs of acid exchanged 1DL products. Note both PMPs and filtered films were finely powdered using a mortar and pestle prior to adding the acidic (10 mM HCl) solution. Exchanged PMPs were imaged, (A) directly after vacuum filtering, and (B) after drying at 80 °C, and films (C) directly after vacuum filtering (D) after drying at 80 °C. In both cases, a substantial change in volume was seen from wet to dry. 13
Figure S12. XRD patterns of finely crushed 1DL film pre- and post-acid exchange. Note low angle peak shifts to higher 2□ after acid exchange which implies a reduction in interplanar spacing from 11.5 Å to 9.5 Å. 14
Figure S13. 1DL gel globules as a result of acidification of 1 g/L 1DL colloidal suspension below the point of zero charge. 15
Figure S14. 1DL gel of 10 g/L colloid formed as the result of titrating with 0.1 M HCl below the point of zero charge. Note: this was a non-quantitative experiment. 15
Figure S15. Titration curve of 1DL suspensions across entire pH range listed in Table S2. Region < pH 6 leads to flocculation, which causes self-buffering behavior and instabilities in pH readings. Blue curve is the system's calculated pH if HCl were added directly with no 1DL in system. Related to data shown in Figure 5B. 16
Figure S16. Evaluation of the linear viscoelastic region (LVER) of 1DL gel-like solid. Storage modulus for the sample with 1.7 mL of acid added (Figure 7). Horizontal (red) line shows 5% drop off in storage modulus, indicating LVER < 4.0%. 16

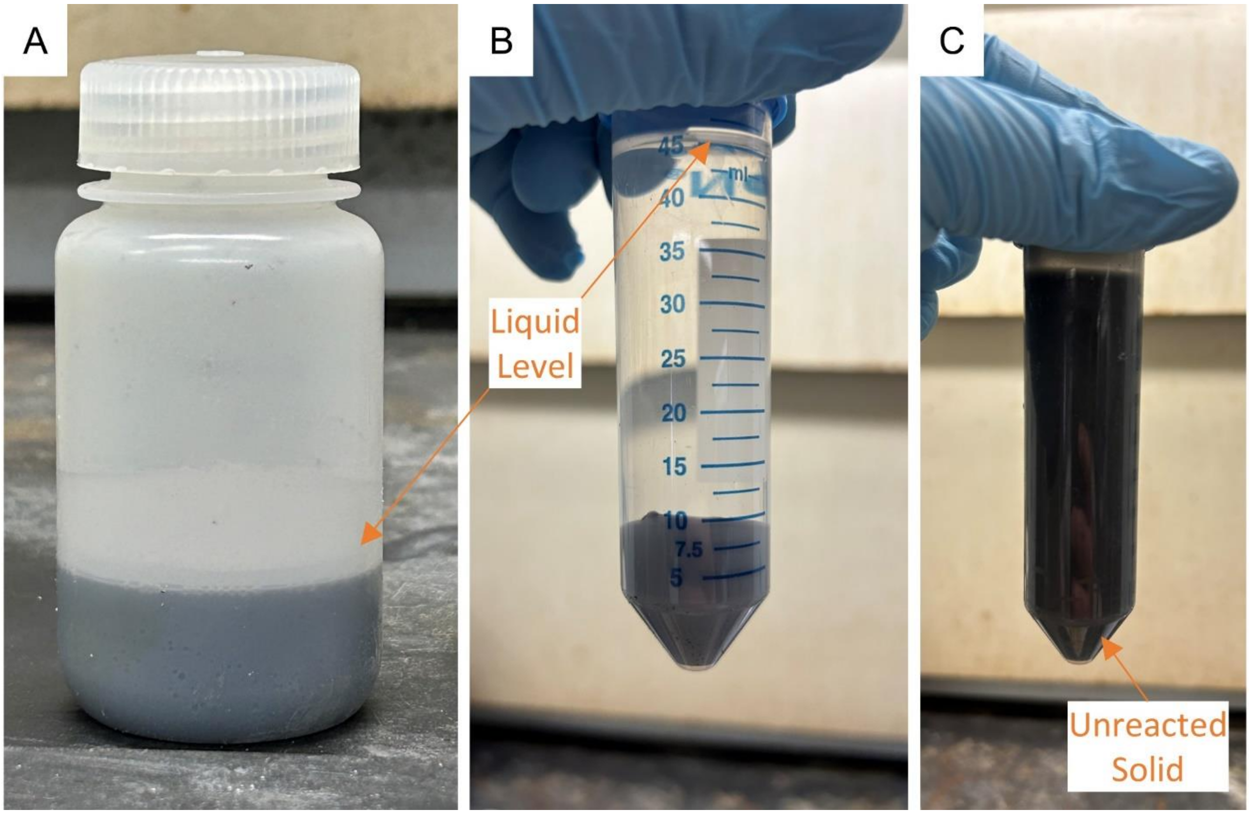

Figure S1. 1DL wash procedure. (A) Slurry of TMAOH and 1DL resulting from the reaction. (B) Post-centrifugation of 1DL slurry shown in (A). (C) 1DL colloid after ethanol washing and suspension in water.

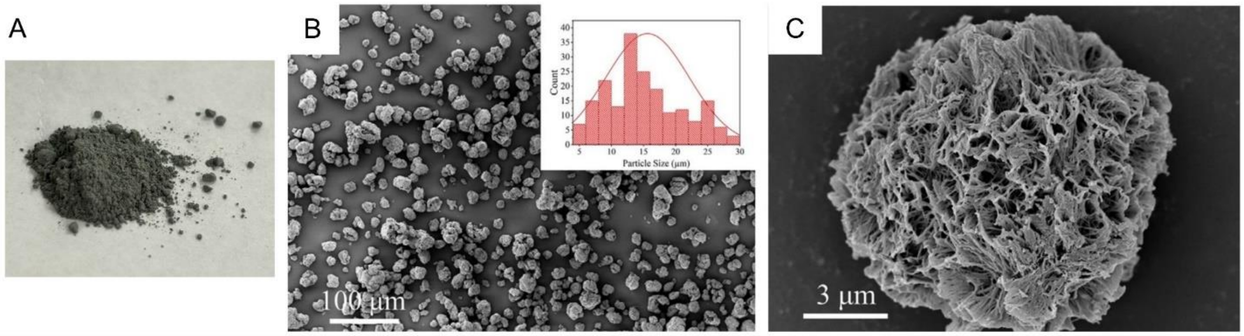

Figure S2. 1DL PMPs. (A) Photograph of 1DL PMP powder. (B) Low-mag SEM micrograph of 1DL PMPs. Inset shows particle size analysis. (C) High-mag SEM micrograph of a single PMP. (B,C) adapted from Badr et al. [1].

Table S1. pH values of acid solutions used in Figure 2. Related to the 1DL acid stability experiment.

Solution	Actual pH	Comments
1		Out of meter range
2	1.94	
3	2.97	
4	4.02	
5	4.92	
6	6.06	Pure fresh DI water

Table S2. Summary of sample compositions used to obtain results shown in Figure 5B and Figure S15. In all cases, colloidal suspension volumes and total volume of mixtures were kept constant. Column 6 lists the final pH values. Last column describes the final product.

Water	HCl (mL)	Colloid (mL)	HCl:Colloid vol./vol.	Total V (mL)	Final pH	Comment
9	0	1	0	10	8.72	Colloid
8.9	0.1	1	0.1	10	8.43	Colloid
8.8	0.2	1	0.2	10	8.34	Colloid
8.7	0.3	1	0.3	10	8.35	Colloid
8.6	0.4	1	0.4	10	8.22	Colloid
8.5	0.5	1	0.5	10	7.54	Colloid
8.4	0.6	1	0.6	10	7.25	Colloid
8.3	0.7	1	0.7	10	7.07	Colloid
8.2	0.8	1	0.8	10	6.47	Colloid
8.1	0.9	1	0.9	10	6.46	Colloid
8	1	1	1	10	6.18	Colloid
7.9	1.1	1	1.1	10	5.99	Colloid
7.8	1.2	1	1.2	10	5.53	Flocced
7.7	1.3	1	1.3	10	3.21	Flocced
7.6	1.4	1	1.4	10	2.97	Flocced
7.5	1.5	1	1.5	10	4.86	Flocced
7	2	1	2	10	3.55	Flocced
6	3	1	3	10	2.54	Flocced

Table S3. Summary of the non-gelled, gel and gel-like solid 1DL products. Photographs of these products are shown in **Figure 6**.

Cation	V added (mL)	Stock [Salt] (M)	Final [Salt] (M)	[1DL] (g/L)	Comments
Time = 15 min					
H^+	0.85	0.1	0.015	8.55	Slight increase in viscosity
Li^+				8.55	
Na^+				8.55	
K^+				8.55	
Mg^{2+}				8.55	Hard
Ca^{2+}				8.55	
Ba^{2+}				8.55	
Fe^{3+}				8.55	Soft
Li^+	0.068	5.0	0.067	9.87	Hard
Na^+	0.17	0.5	0.015	9.67	Soft
Time = 1 week					
Na^+	1.7	0.1	0.025	7.46	Slight increase in viscosity
H^+	0.85		0.015	8.55	Slight increase in viscosity
Li^+				8.55	Soft
Na^+				8.55	Slight increase in viscosity
K^+				8.55	Very soft
Mg^{2+}				8.55	Soft
Ca^{2+}				8.55	Floc formation
Ba^{2+}				8.55	Hard, detach from wall of vial
Fe^{3+}				8.55	Floc formation
H^+	0.43		0.008	9.21	Soft
Li^+				9.21	Slight increase in viscosity
Na^+				9.21	
K^+				9.21	
Mg^{2+}				9.21	
Ca^{2+}				9.21	Soft, detach from wall of vial
Ba^{2+}				9.21	Soft
Fe^{3+}				9.21	Hard, detach from wall of vial
H^+	0.28		0.005	9.47	No noticeable change in viscosity
Li^+				9.47	
Na^+				9.47	
K^+				9.47	
Mg^{2+}				9.47	Slight increase in viscosity
Ca^{2+}				9.47	Soft, detach from wall of vial
Ba^{2+}				9.47	Soft
Fe^{3+}				9.47	Hard, detach from wall of vial

SI Section 1 – Wash Solvent Effects on 1DL PMPs

To understand the impact of solvent on the 1DL washing process various alcohols were explored: methanol, ethanol, propanol, isopropanol, butanol, and tert-butanol. All were obtained as pure solvents, without water, and used as-received. 10 mL of 1DL slurry (**Figure S1A**) was centrifuged and the residual TMAOH was poured off. Then ≈ 35 mL of each solvent was added to the damp sediment. The mixtures were combined via vortex mixing then centrifuged to separate the solid from the extract that was poured off each time. This procedure was repeated 3x – which is the standard process for 1DL washing with ethanol.

Each solvent had a unique interaction with the 1DL solid – seemingly based on their polarity and water miscibility. **SI Video 1** shows the mixtures of the 1DL solid and the alcohols during washing. It is apparent that only methanol and ethanol are sufficiently miscible with the mixture. The propanol and isopropanol produce clumped solids. Butanol and tert-butanol are not miscible at all and create mixtures similar to oil and water. In this reaction, using 25 wt.% TMAOH, there is substantial left-over reactant thus the washing step is used to both remove residual OH^-, but also TMA^+. We believe the solvent miscibility is related to the amount of TMA^+ (a highly hydrophilic cation) left over after washing.

Based on the TGA results shown in **Figure S3A,** the effectiveness of each wash solvent is related to the amount of residual water and TMA^+ left in the system. The first event for each of the samples is related to the loose solvent loss, which occurs at < 120 °C. According to our earlier work [2], the mass loss before ≈ 250 °C is related to water loss, the first event being weakly bound and the second being more strongly bound water. It is

difficult to determine without further analysis if the mass loss (and residual liquid shown in **Figure S4-9 top left**) for all samples except methanol and ethanol is solely due to water, or a mixture of water, solvent, and *non-bound* TMA^+. In our experience, it is more probably the latter. In the 250 °C to 400 °C temperature range, normalized to the weight at 250 °C, is shown in **Figure S3B.** From this result, it is reasonable to conclude this mass loss is related to the structurally bound TMA^+. Importantly, the amount of bound TMA^+ is consistent across all wash solvents, implying that the interlayer space is devoid of solvent.

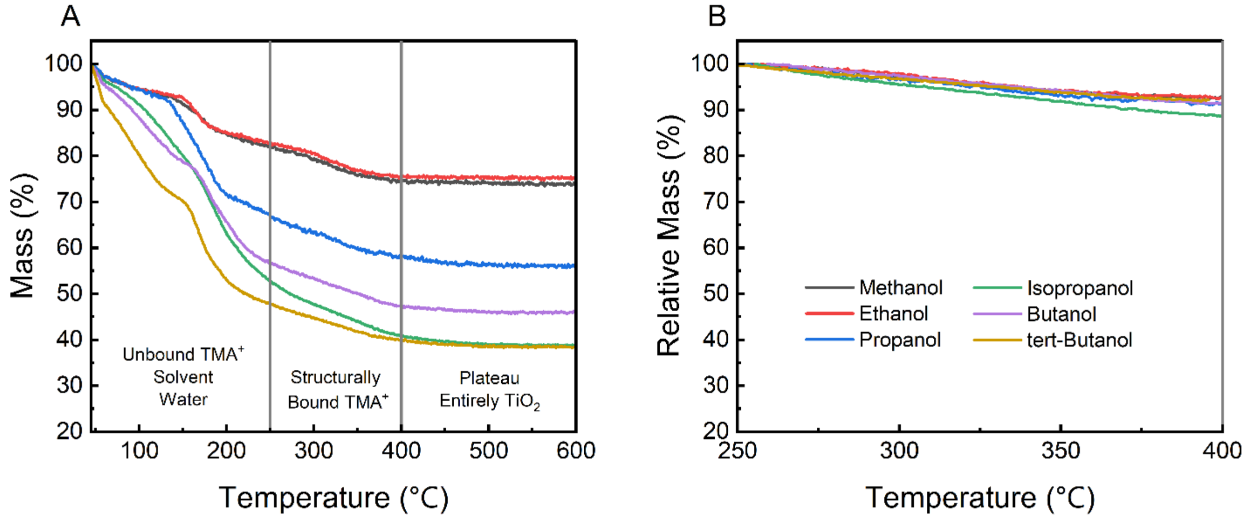

Figure S3. TGA analysis of 1DL PMPs formed by washing with various solvents indicated on figure. Samples were dried at RT under vacuum prior to analysis. (A) TGA data for all solvents. Raw TGA data was normalized at 45 °C. (B) Relative mass loss from 250 °C to 400 °C for each solvent, corresponding to the region of structurally bound TMA^+. Curves are color coordinated to legend in (B).

To ensure the powders were sufficiently dried to image in an SEM, the materials shown in **Figure S4-9** were dried under vacuum at 100 °C, which was enough to remove the residual solvent as well as the free water and TMA^+. The resulting morphologies varied. The methanol product (**Figure S4**) produces agglomerated sheets of 1DL solids, similar to those in our previous work on self-assembled 1DL suspensions[3]. The EtOH

product (**Figure S5**) is identical to the PMPs (**Figure S2**) [1], as expected. The propanol (**Figure S6**) and butanol (**Figure S8**) solids are similar in their morphologies, showing similar porous products to EtOH (**Figure S5**), but slightly more agglomerated. Washing with isopropanol (**Figure S7**) and tert-butanol (**Figure S9**) produces particles, unlike methanol (**Figure S4**), that appear to be substantially less porous than those formed with EtOH (**Figure S5**).

Although these results do not support a shift from EtOH as the primary wash solvent of choice, they do reinforce the initial choice of EtOH in our early work [4, 5]. However, these results show novel insight into the assembly and interactions of 1DLs with solvents and showcase the highly reactive nature of their surfaces – so reactive that a simple change in solvent system causes significant morphological changes at the micron scale.

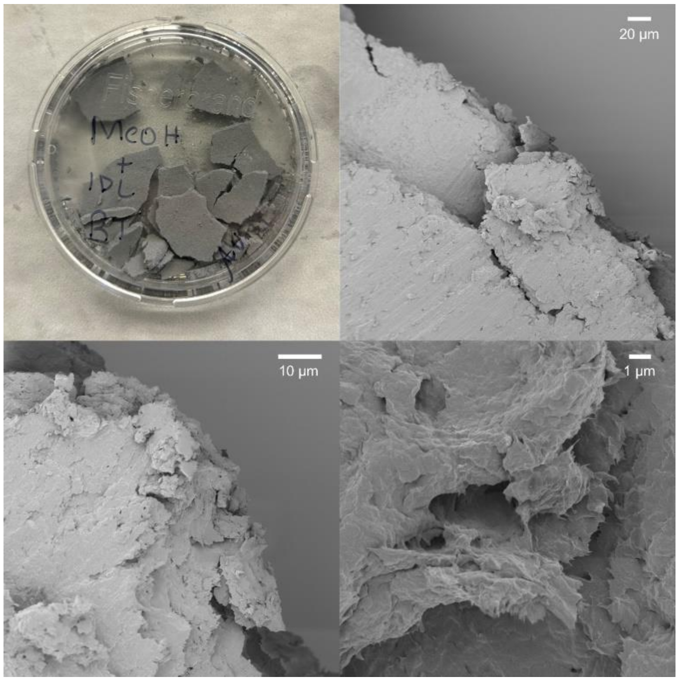

Figure S4. Methanol-washed 1DL product. Photograph of RT dried product and SEM micrographs of the products dried at 100 °C at various magnifications.

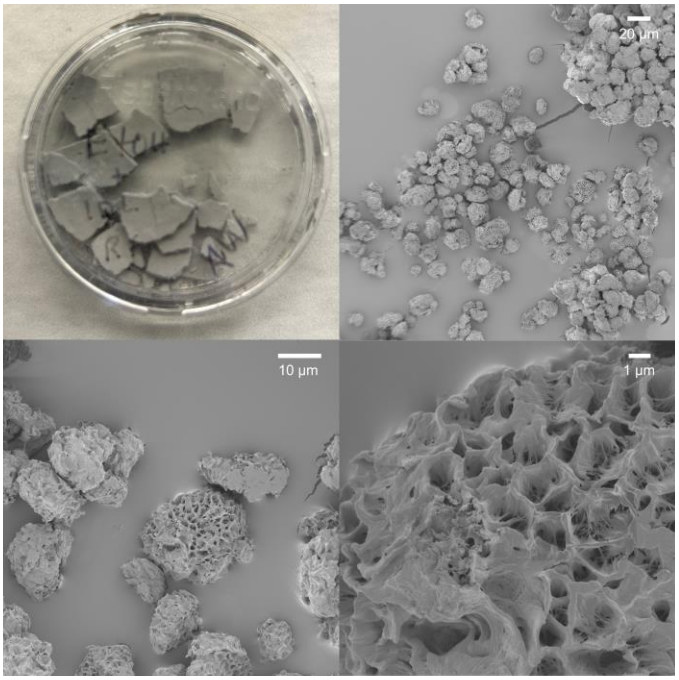

Figure S5. Ethanol-washed 1DL product. Photograph of the RT dried product and SEM micrographs of the product dried at 100 °C at various magnifications.

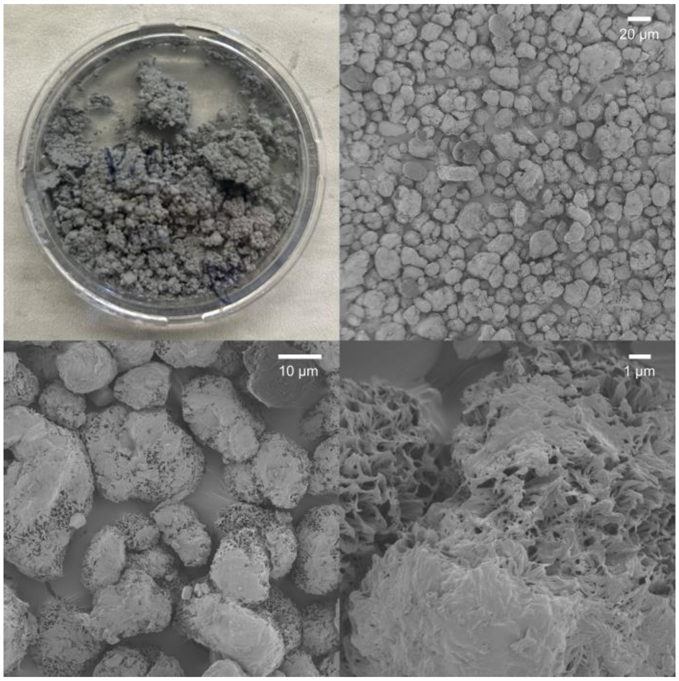

Figure S6. Propanol-washed 1DL product. Photograph of the RT dried product and SEM micrographs of the product dried at 100 °C at various magnifications.

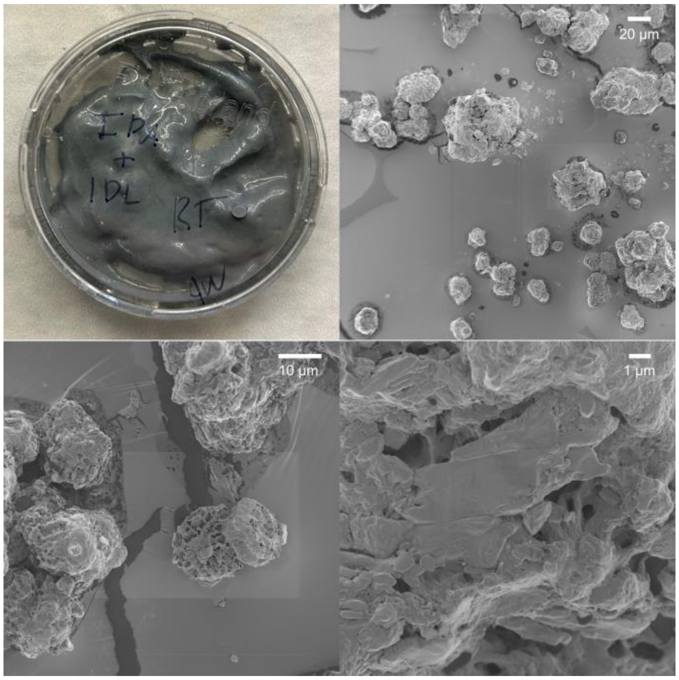

Figure S7. Isopropanol-washed 1DL product. Photograph of RT dried product and SEM micrographs of the product dried at 100 °C at various magnifications. Note residual liquid in the photograph.

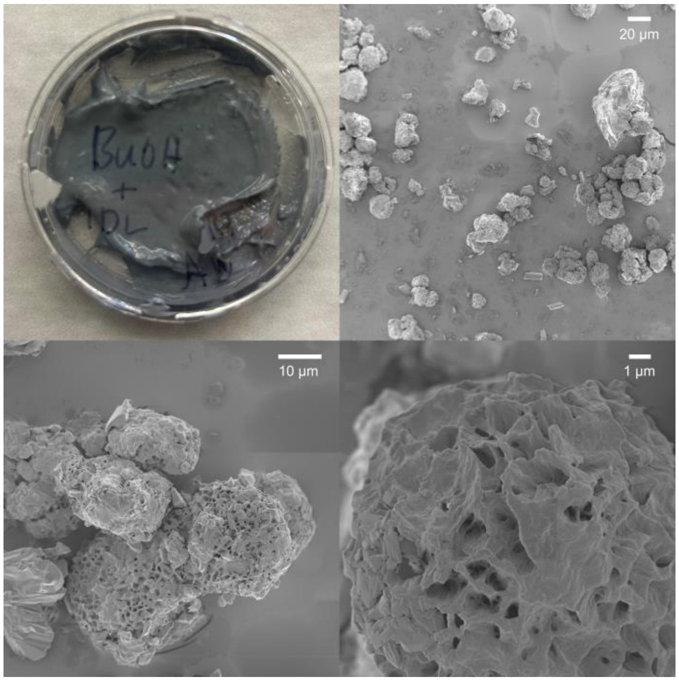

Figure S8. Butanol-washed 1DL product. Photograph of RT dried product and SEM micrographs of product dried at 100 °C at various magnifications. Note residual liquid in photograph.

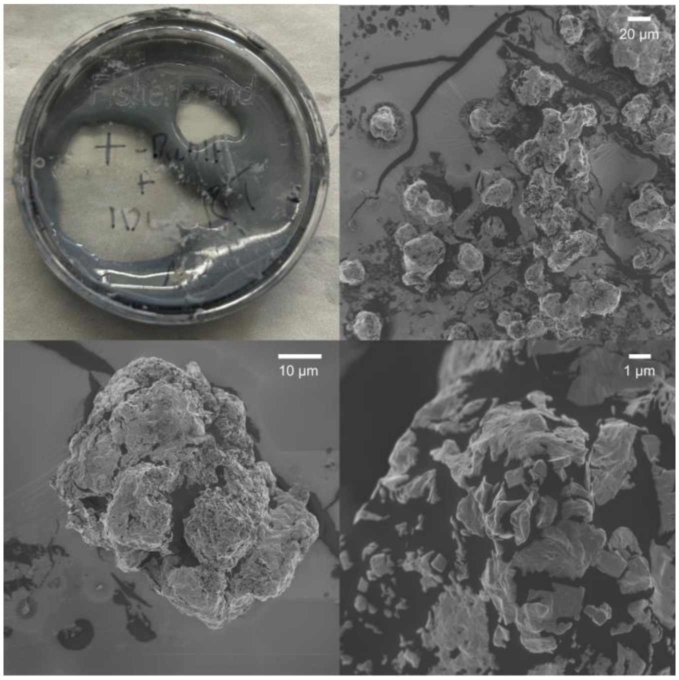

Figure S9. Tert-Butanol-washed 1DL product. Photograph of RT dried product and SEM micrographs of the product dried at 100 °C at various magnifications. Note residual liquid in photograph.

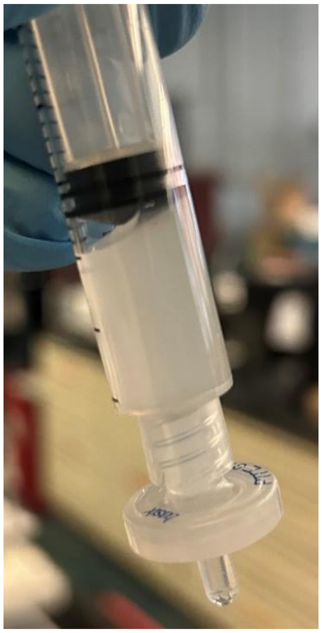

Figure S10. Re-suspended 1DL particles in water. Filter is a < 0.45 μm PTFE syringe filter. Re-suspended particles resist filtration through such filters.

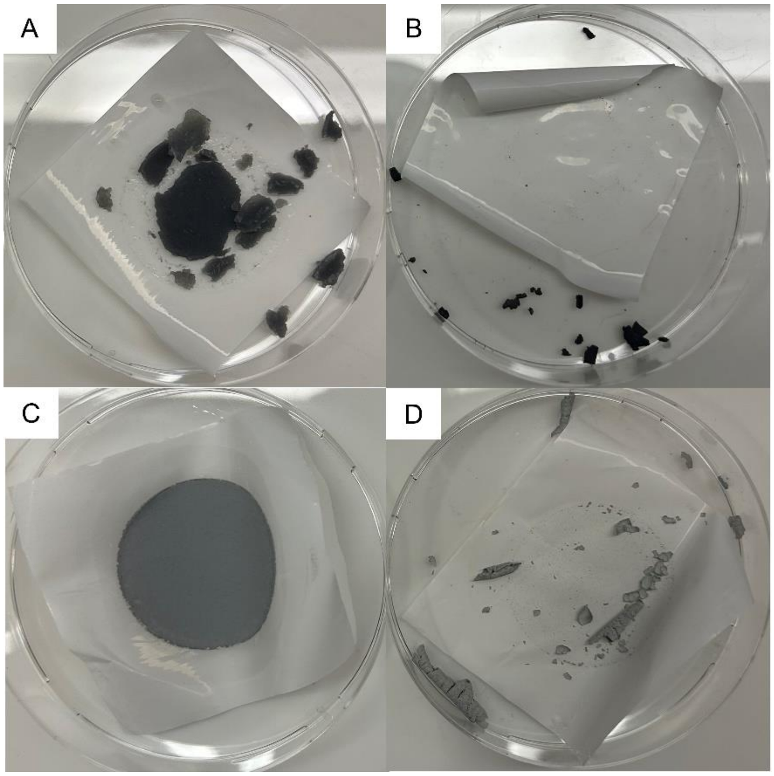

Figure S11. Photographs of acid exchanged 1DL products. Note both PMPs and filtered films were finely powdered using a mortar and pestle prior to adding the acidic (10 mM HCl) solution. Exchanged PMPs were imaged, (A) directly after vacuum filtering, and (B) after drying at 80 °C, and films (C) directly after vacuum filtering (D) after drying at 80 °C. In both cases, a substantial change in volume was seen from wet to dry.

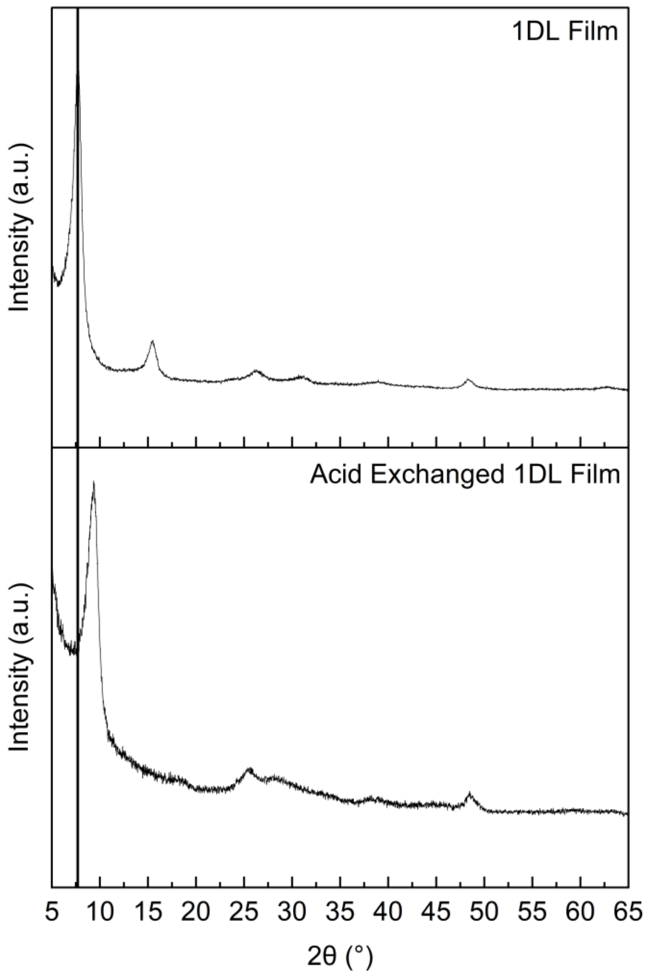

Figure S12. XRD patterns of finely crushed 1DL film pre- and post-acid exchange. Note low angle peak shifts to higher 2q after acid exchange which implies a reduction in interplanar spacing from 11.5 Å to 9.5 Å.

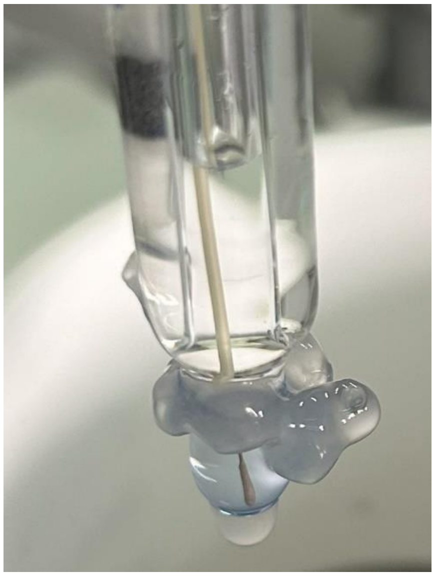

Figure S13. 1DL gel globules as a result of acidification of 1 g/L 1DL colloidal suspension below the point of zero charge.

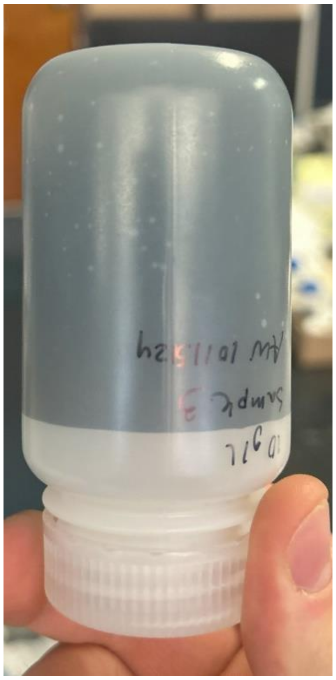

Figure S14. 1DL gel of 10 g/L colloid formed as the result of titrating with 0.1 M HCl below the point of zero charge. Note: this was a non-quantitative experiment.

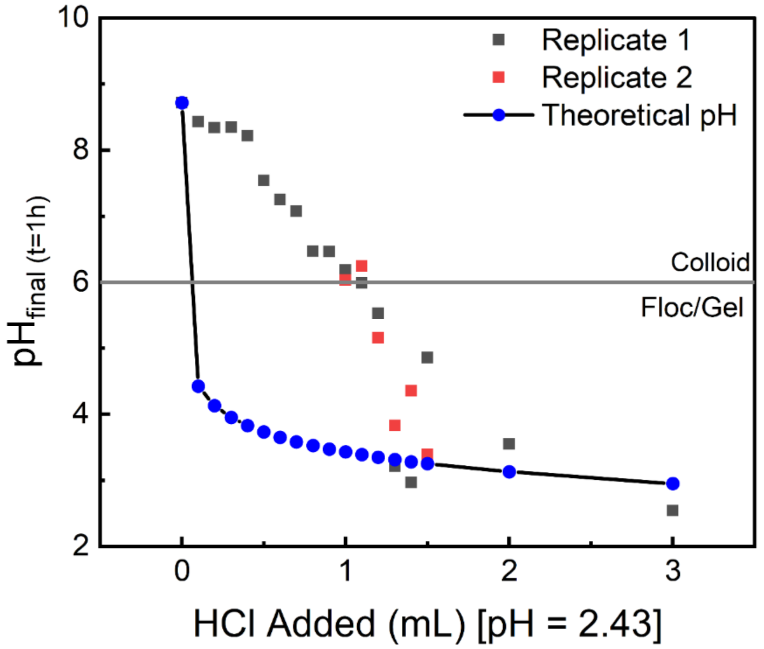

Figure S15. Titration curve of 1DL suspensions across entire pH range listed in Table S2. Region < pH 6 leads to flocculation, which causes self-buffering behavior and instabilities in pH readings. Blue curve is the system's calculated pH if HCl were added directly with no 1DL in system. Related to data shown in **Figure 5B**.

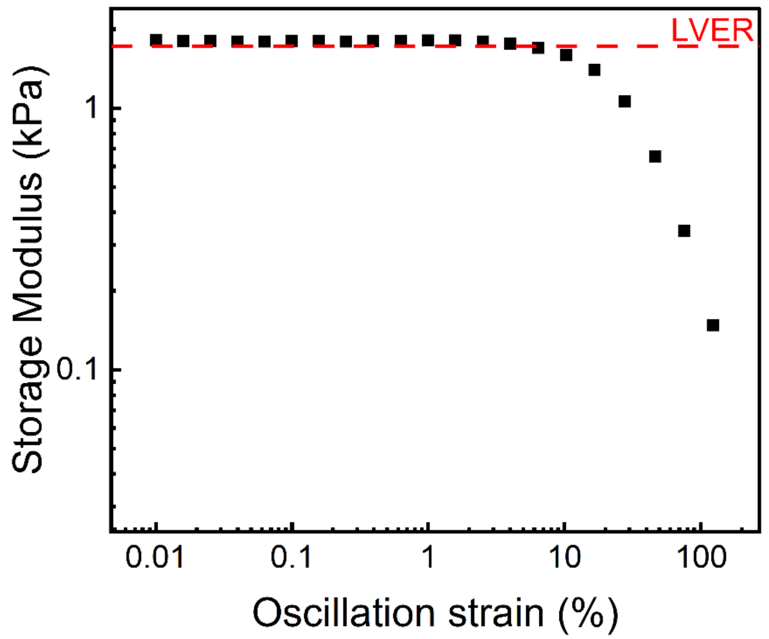

Figure S16. Evaluation of the linear viscoelastic region (LVER) of 1DL gel-like solid. Storage modulus for the sample with 1.7 mL of acid added (**Figure 7**). Horizontal (red) line shows 5% drop off in storage modulus, indicating LVER < 4.0%.

Supplemental References

(1) Badr, H. O.; Cope, J.; Kono, T.; Torita, T.; Lagunas, F.; Castiel, E.; Klie, R. F.; Barsoum, M. W. Titanium oxide-based 1D nanofilaments, 2D sheets, and mesoporous particles: Synthesis, characterization, and ion intercalation. *Matter* **2023**, *6* (10), 3538-3554. DOI: 10.1016/j.matt.2023.07.022.

(2) Mieles, M.; Walter, A. D.; Wu, S.; Zheng, Y.; Schwenk, G. R.; Barsoum, M. W.; Ji, H.-F. Hydronium-Crosslinked Inorganic Hydrogel Comprised of 1D Lepidocrocite Titanate Nanofilaments. *Advanced Materials* **2024**, 2409897. DOI: https://doi.org/10.1002/adma.202409897.

(3) Schwenk, G. R.; Walter, A. D.; Barsoum, M. W. Solvent-Driven Self-Assembly of One-Dimensional Lepidocrocite Titanium-Oxide-Based Nanofilaments. *Nano Lett.* **2024**, *24* (25), 7584-7592. DOI: 10.1021/acs.nanolett.4c00921.

(4) Badr, H. O.; El-Melegy, T.; Carey, M.; Natu, V.; Hassig, M. Q.; Johnson, C.; Qian, Q.; Li, C. Y.; Kushnir, K.; Colin-Ulloa, E.; et al. Bottom-up, scalable synthesis of anatase nanofilament-based two-dimensional titanium carbo-oxide flakes. *Materials Today* **2022**, *54*, 8-17. DOI: 10.1016/j.mattod.2021.10.033.

(5) Badr, H. O.; Lagunas, F.; Autrey, D. E.; Cope, J.; Kono, T.; Torita, T.; Klie, R. F.; Hu, Y.-J.; Barsoum, M. W. On the structure of one-dimensional TiO2 lepidocrocite. *Matter* **2022**, *6* (1), 128-141. DOI: https://doi.org/10.1016/j.matt.2022.10.015.
